# Electrochemically detecting DNA methylation in the *EN1* gene promoter: implications for understanding ageing and disease

**DOI:** 10.1042/BSR20202571

**Published:** 2020-11-17

**Authors:** Amy E. Morgan, Katie D. Acutt, Mark T. Mc Auley

**Affiliations:** Faculty of Science and Engineering, University of Chester, Thornton Science Park, Pool Lane, Chester, CH2 4NU, U.K.

**Keywords:** Aging, biosensor, electrochemistry, methylation

## Abstract

There is a growing need for biomarkers which predict age-onset pathology. Although this is challenging, the methylome offers significant potential. Cancer is associated with the hypermethylation of many gene promoters, among which are developmental genes. Evolutionary theory suggests developmental genes arbitrate early-late life trade-offs, causing epimutations that increase disease vulnerability. Such genes could predict age-related disease. The aim of this work was to optimise an electrochemical procedure for the future investigation of a broad range of ageing-related pathologies. An electrochemical approach, which adopted three analytical techniques, was used to investigate DNA methylation in the engrailed-1 (*EN1*) gene promoter. Using synthetic single-stranded DNA, one technique was able to detect DNA at concentrations as low as 10 nM, with methylation status distinguishable at concentrations >25 nM. A negative correlation could be observed between % methylation of a heterogeneous solution and the key electrochemical parameter, charge transfer resistance (R_ct_; r = −0.982, *P*<0.01). The technique was applied to the breast cancer cell line Michigan Cancer Foundation-7 (MCF-7), where a similar correlation was observed (r = −0.965, *P*<0.01). These results suggest electrochemistry can effectively measure DNA methylation at low concentrations of DNA. This has implications for the future detection of age-related disease.

## Introduction

Ageing is a complex biological phenomenon which is characterised by a decline in physical function [1]. This physical deterioration results in an increased risk of a multitude of age-related diseases, such as cardiovascular disease (CVD), Alzheimer’s disease (AD) and cancer [2]. These diseases place a significant burden on the wellbeing of older people. As an example of this morbidity, almost half of American adults (48%) currently live with CVD [3]. Moreover, from a global perspective, 60% of all cancer cases and 70% of cancer-related deaths occurs in older people (those ≥65 years) [4]. Based on this evidence it is cogent that appropriate biomedical tools are developed to help diagnose and predict age-associated diseases so the healthspan of older people can be extended. Due to its dense tapestry, which involves a multitude of perturbations at the molecular, biochemical and cellular levels, it is not straightforward to select a biomarker of ageing which can be reliably used to predict age-related disease, with a high degree of accuracy in humans [5,6]. However, experimental and computational evidence in the last decade has strongly suggested that DNA methylation changes can act as a powerful marker of ageing [7–12]. Specifically, advancing age is often accompanied by global hypomethylation, as outlined by Barciszewska et al. (2007) who used a TLC-based detection method to examine global age-related hypomethylation in human skin fibroblasts [13]. Global hypomethylation is observed in conjunction with site-specific hypermethylation at the promoter region of a variety of genes, which have been ubiquitously associated with age-related disease [14]. Among the genes whose methylation profile can change with age are developmental genes. The aberrant methylation of developmental genes has regularly been associated with disease [15,16]. Given that ageing can be defined from an evolutionary point of view as the decline in the force of natural selection with age [17–19], it is natural to infer that alterations to DNA methylation patterns among developmental genes is the consequence of an early-late life trade-off. This line of thinking aligns with the mutation accumulation [18] or antagonistic pleiotropy theory of ageing [19], and offers the possibility that methylation changes at developmental genes could in part mediate the effects of deleterious age-specific epimutations which increase our susceptibility to disease over time, as these mutations gradually accumulate in the genome. It is also worth noting that the disposable soma theory (DST) [20], which is a physiological version of the antagonistic pleiotropy theory is based on the assumption that there is a trade-off between reproductive investment and somatic maintenance could also account for age-related DNA methylation changes in gene promoters.

Based on our evolutionary logic, it is clear that age-related methylation changes to developmental genes could have the potential to act as a predictor of age-related diseases. There are many developmental genes which could be investigated for their predictive capabilities, however due to its strong association with cancer, the engrailed-1 (*EN1*) gene is an excellent candidate. The *EN1* gene plays an important role in pattern formation during embryonic development. First identified in *Drosophilia*, mutations to the homoeobox gene have been associated with abnormal development; specifically, posterior-anterior duplications and wing malformations. In mice, the *En1* gene has been associated with limb innervation [21], and cerebellum patterning, with En1-null mice failing to form a cerebellum and incurring perinatal death [22]. The human orthologue EN1 has been associated with central nervous system pattern formation, with expression observed in the midgestational medulla and cerebellum [23]. Despite this gene playing a vital role during embryonic development, EN1 has been associated with the development of cancer later in life, through changes to the methylation patterns within the gene promoter. Hypermethylation of the EN1 promoter has been associated with a reduced gene expression in colorectal [24], prostate [25] and lung cancer [26]. Additionally, several studies have correlated *EN1* gene hypermethylation with tumour size/grading and mortality [27,28]. Moreover, the importance of EN1 has been underscored due to its role in protecting mesencephalic dopaminergic neurons from oxidative stress, in a mouse model of Parkinson’s disease [29]. Thus, these genes are important to ageing and the methylation of the EN1 gene promoter offers a potential biomarker for prediction of age-associated conditions, and in particular cancer.

The aim of this work is to use the EN1 promoter as a model for quantifying methylation. To do this, there a number of traditional experimental techniques which could be adopted. These include bisulphite sequencing, methylation-specific PCR and microarray analysis [30,31]. Unfortunately, these techniques are time-consuming and expensive, and do not make ideal platforms for the future development of biosensors which are capable of rapidly, efficiently and cheaply predicting the onset of age-related disease. In contrast, electrochemical methods help to overcome these challenges, and in recent years have been successfully used to quantify gene promoter methylation [32–34]. Most notably, the utility of this approach with the EN1 promoter has been underscored by the work of Koo et al. who in recent years introduced the eMethylsorb method [35–37]. This procedure involves adsorbing bisulphite-treated and asymmetrically amplified DNA on to a gold electrode before submerging the electrode into Fe^2+^/Fe^3+^ redox solution, within a three-electrode redox cell, and conducting differential pulse voltammetry (DPV). Bisulphite treatment converts unmethylated cytosines into uracil, while methylated cytosines remain unchanged [38]. Asymmetric PCR then produces single-stranded DNA rich in either adenine or guanine [39]. As nucleotides bind to gold with the following affinities: A > C ≥ G > T [40], treated unmethylated samples passivate the surface to a greater degree than methylated samples. This is then observed as a differential electrochemical signal. Using this principle, synthetic 30 base ssDNA, designed to represent methylated and unmethylated variants of a region downstream of the transcription start site of the EN1 gene promoter, that have undergone bisulphite treatment and asymmetric amplification, will be adsorbed to a gold rotating disk electrode (Au-RDE). Electrochemical impedance spectroscopy (EIS) and cyclic voltammetry (CV) measurements will be taken, in addition to DPV. After optimisation, the procedure will be applied to bisulphite-treated and asymmetrically amplified DNA extracted from the breast cancer cell line, Michigan Cancer Foundation-7 (MCF-7).

## Materials and methods

### Synthetic oligonucleotides

Synthetic oligonucleotides were used to represent methylated and unmethylated variants of a region downstream of the transcription start site, of the EN1 gene promoter, which had undergone bisulphite treatment and asymmetric amplification (Table 1). The oligonucleotides were 30 bases in length, and contained six cytosine-phosphate-guanine (CpG) sites (Eurofins Genomics). Samples were diluted to 50 nM in 1× phosphate-buffered saline (PBS; 137 mM sodium chloride, 2 mM potassium chloride, 10 mM phosphate buffer, pH 7.4, Amresco, E404-200TABS) for the optimisation of adsorption time and rotation speed. To examine the effect of concentration, a range of 0–400 nM was employed. Following this test, 200 nM solutions were used to detect % methylation. Solutions were stored at 4ºC for 1 month, and were tested at room temperature.

**Table 1:** Synthetic ssDNA sequences

Oligonucleotide	5′-sequence-3′
Methylated sequence	GATAACGACGACAATAAAAACGACGCGAAA
Unmethylated sequence	AATAACAACAACAATAAAAACAACACAAAA

CpG sites are underlined.

### Cell culturing

MCF-7 cells (gifted from, and grown at, Chester Medical School, University of Chester) were cultured in Eagle’s Minimum Essential Medium (Lonza, LZBE12-611F) supplemented with 10% foetal bovine serum (Invitrogen Gibco, Fisher, 11573397) and 1% non-essential amino acids (SLS, M7145), and incubated at 37°C at 5% CO_2_. Cells were trypsinised at 70% confluence for DNA extraction.

### DNA extraction and preparation

DNA was extracted from MCF-7 cells using QIAGEN QIAamp® DNA Mini Kit [50] (QIAGEN, 51304) according to the manufacturer’s instructions. Extracted DNA was stored at −20ºC. After dilution in nuclease-free water (USB Corporation, 71786), the concentration of DNA was calculated as 739.8 μg/ml using a Varioskann Lux fluorescent plate reader (Thermo Scientific), using the equation:

$$\text{Concentration (µg}/\text{ml)}={\text{ (A}}_{260}{\text{ - A}}_{320}\text{) }\times \;\text{ Dilution factor }\times \;\;50\;\text{µg}/\text{ml}$$

Purity was calculated as 1.763 via the equation:

$$\text{DNA}\;\text{purity}=\frac{{\text{A}}_{260}}{{\text{A}}_{280}}$$

Whole genome DNA (Roche Diagnostics GmbH, 11691112001) was diluted in nuclease-free water to a concentration of 50 ng/μl and amplified using the whole genome amplification kit – REPLI-g UltraFast Mini kit (QIAGEN, 150033) according to the manufacturer’s instructions. It was estimated that a concentration of 500 ng/μl was created. The MCF-7 DNA and whole genome amplified (WGA) DNA then underwent bisulphite modification using the MethylEasy Xceed kit (Human Genetic Signatures, ME002) according to the manufacturer’s instructions, to produce 20 ng/μl bisulphite-modified DNA. The bisulphite-modified DNA was stored at −20°C.

### Asymmetric PCR and amplification verification

Asymmetric PCR was utilised to generate ssDNA of a section of the EN1 gene promoter from MCF-7 and WGA DNA. For the first round of PCR, 2 μl of 20 ng/μl MCF-7 or WGA DNA was combined with 12.5 μl of 2× PCR master mix (Bio-Rad, 1662119), 6.5 μl nuclease-free water, 3 μl of 10 μM forward primer, and 1 μl of 1 μM reverse primer (Eurofins Genomic, 30:1 primer ratio). For the second round of PCR, 2 μl of PCR product was combined with 12.5 μl of 2× PCR master mix, 4.5 μl nuclease-free water, 5 μl of 10 μM forward primer, and 1 μl of 1 μM reverse primer. Primer ratio for second round of PCR was 50:1 as outlined in Table 2 [39]. The thermocycler was programmed for predenaturation at 95°C for 3 min, before a 30-cycle program of denaturation at 95°C for 1 min, annealing at 50°C for 2 min, and extension at 72°C for 2 min. Final extension followed for 10 min at 72°C. Programming was constant for first and second round PCR. Secondary PCR product was stored for 1 week at 4°C.

**Table 2:** Asymmetric PCR forward and reverse primers

Primer	5′-sequence-3′	Concentration (μM)	
		First round	Second round
Forward	ATTCAGTCCACAACAAYGTTGGTTGAGTTTATAA GTAGGATAGT	1.2	2
Reverse	ACRACCRCAACAACCAAACCCT	0.04	0.04

To verify amplification, gel electrophoresis was conducted on 2% agarose gel containing 1× GelRed nucleic acid stain (Biotium, 41003-1) in 1× TAE buffer (Bio-Rad, 166-0742). Samples were prepared by combining 10 μl of Orange G loading buffer (Bio-Rad, 1662119) with 25 μl of secondary PCR product before 10 μl of sample was added to each loading well. Gel electrophoresis was run at 100 V for 90 min, and images were taken using UVP BioDoc-It 220 Imaging system M-20V Transilluminator and Doc-It LS image analysis software (version 8.6).

For electrochemical testing, 600 μl (1/3), 100 μl (1/18), 50 μl (1/36), and 10 μl (1/180) of secondary PCR product were combined with 1× PBS to make an overall volume of 1.8 ml. A dilution of 1/18 was used to test % methylation in heterogeneous samples of MCF-7 and WGA DNA (0, 25, 50, 75, and 100%).

### Electrochemical testing

The redox cell consisted of: 2 mm Au-RDE (Radiometer analytical, BM-EDI101), Ag/AgCl reference electrode (ALS, RE1CP), 0.127 mm diameter coiled platinum counter electrode (Alfa Aesar, 00263), in ∼70 ml of 2.5 mM Fe^2+^/2.5 mM Fe^3+^/1× PBS redox solution (Potassium hexacyanoferrate (II) trihydrate, AnalaR NORMAPUR, 26816.298, and potassium hexacyanoferrate (III), AnalaR NORMPUR, 26810.232) containing a magnetic stirring bar. The redox cell was placed on a plastic topped magnetic stirring platform (HANNA Instruments, HI-190M), and electrodes were attached to the potentiostat (Princeton Applied Research, BiStat). The potentiostat was controlled via the software EC-Lab V11.10.

**Figure 1:**
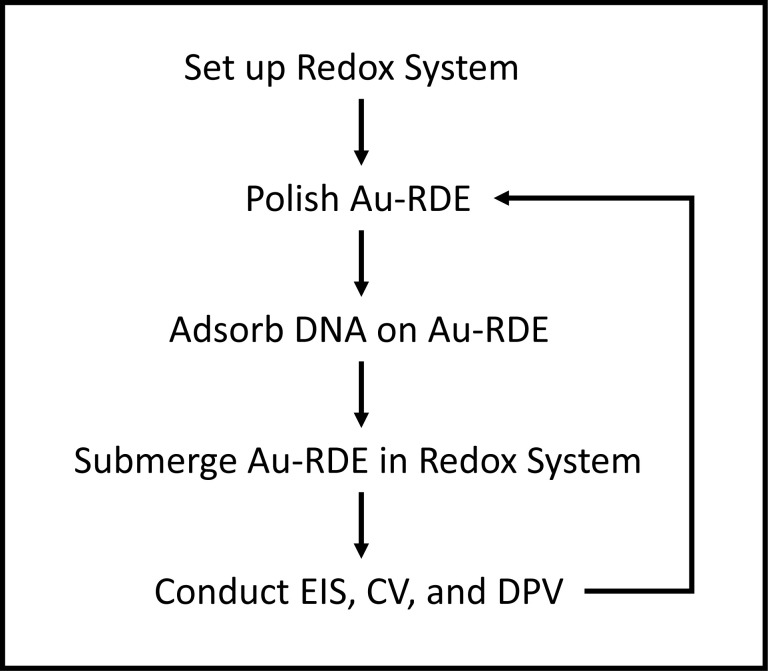
Overview of electrochemical procedures

Before each adsorption step, the Au-RDE electrode was polished using figure of eight motions for 30 s on silk (Kemet, 341752) with 6-μm diamond spray, on silk with 3-μm diamond spray (Kemet, 116004), and on felt (Kemet, 341208) with a saturated 1-μm alumina solution (Kemet, 600253). Between each step, the electrode was rinsed in ultrapure water and dried. Following this three-step polishing process, the electrode was sonicated in ultrapure water for 30 s, and dried. Following polishing, DNA samples were adsorbed on to the Au-RDE at an appropriate rotation speed for an appropriate time, before insertion into the redox cell. The redox solution was mixed for 10 s before each EIS, CV, and DPV measurement was taken (Figure 1).

EIS was conducted at open circuit potential, at a scanning frequency between 200 kHz and 100 mHz, with 10 points per decade and a voltage amplitude of 20 mV. A Z-fit analysis was conducted within EC-Lab V11.10, using the equivalent circuit selection R1 + Q2/(R2 + W2) to gain parameter values for R2. R2 was subsequently referred to as charge transfer resistance (R_ct_). CV was performed at open circuit potential with a scan speed of 200 mV/s up to a vertex potential of 0.8 V and backwards to −0.15 V vs. Ag/AgCl with a potential step of 1 mV, and peak to peak separation (Δ*E_p_*). From the cyclic voltammogram, *ΔEp* (mV) was determined. DPV was performed by scanning the potential between −0.2 and 0.7 V vs. Ag/AgCl with a potential step of 5 mV, a pulse amplitude of 50 mV, a pulse width of 50 ms, and a pulse period of 100 ms [41]. Peak anodic current, *i_pa_* (μA) was recorded from the differential pulse voltammogram. Results recorded as mean ± 1 standard deviation. RSD, also known as the coefficient of variance, was also reported. Correlations were evaluated using a Pearson’s correlation analysis on SPSS Version 25. To undertake multiple comparisons analyses, a one-way ANOVA with a Tukey’s post-hoc test was conducted.

## Results

### EIS

#### Synthetic oligonucleotides

**Figure 2:**
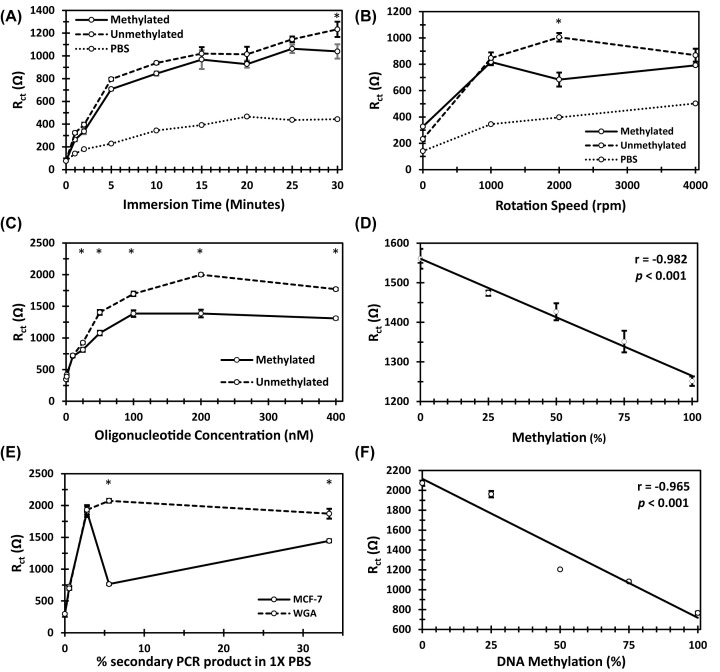
Influence of DNA methylation on R_ct_ R_ct_ determined through Z-fit analysis of Nyquist plots for a 2-mm Au-RDE in 2.5 mM ferrocyanide/2.5 mM ferricyanide/1× PBS after adsorption of ssDNA. Optimisation of (**A**) adsorption time of 50 nM ssDNA at a rotational speed of 2000 rpm, (**B**) rotation speed of Au-RDE for adsorption of 50 nM ssDNA for 5 min, and (**C**) oligonucleotide concentration adsorbed at 2000 rpm for 5 min. (**D**) Depicts the correlation between % methylation of heterogeneous samples of 200 nM synthetic oligonucleotides adsorbed on to the Au-RDE for 5 min at 200 rpm, and R_ct_. Pearson’s linear correlation coefficient r, and *P* are displayed on plot. (**E**) Optimisation of amount of secondary PCR product, derived from bisulphite-treated MCF-7 and WGA DNA, in 1.8 ml of 1× PBS, adsorbed on to Au-RDE for 5 min at 2000 rpm. Results correspond to 1/180, 1/36, 1/18 and 1/3 dilutions. (**F**) Depicts the correlation between % methylation of heterogeneous samples of 1/18 MCF-7 (methylated) and WGA (unmethylated) DNA adsorbed on to Au-RDE for 5 min at 2000 rpm, and R_ct_. Pearson’s linear correlation coefficient r, and *P* are displayed on plot. * indicates *P*<0.05 between methylated and unmethylated DNA in a one-way ANOVA statistical analysis.

The first variable to be optimised was DNA adsorption time (Figure 2A). A DNA adsorption time of 1 min was sufficient to bring about a statistically significant increase in R_ct_, when compared with 0 min (*P*<0.05). However, it was not until 30 min that R_ct_ was distinguishable between 50 nM methylated and unmethylated samples (*P*<0.05). The second variable optimised was rotation speed (Figure 2B). Z-fit analysis of electrochemical impedance spectra revealed the greatest difference in R_ct_ between 50 nM methylated and unmethylated samples was at a rotation speed of 2000 rpm, with a difference of 321.43 Ω between means. Moreover, the R_ct_ for 50 nM methylated and unmethylated DNA samples were exclusively statistically different at a rotation speed of 2000 rpm (*P*<0.05). Electrochemical impedance spectra and data can be observed in the supplementary results file, Appendix A. The third parameter to be optimised was DNA concentration (Figure 2C). The limit of detection was 10 nM for both methylated and unmethylated DNA, as determined by a one-way ANOVA with a Tukey’s post-hoc test. Importantly, methylation status was not distinguishable until >25 nM (*P*<0.05). However, the greatest difference between methylated and unmethylated DNA solutions was observed at 200 nM. Therefore, 200 nM was determined as the optimum concentration to determine DNA methylation, and was the concentration of choice to detect % methylation in a heterogeneous sample of methylated and unmethylated DNA (Figure 2D). A strong negative correlation between % methylation and R_ct_ was observed (r = −0.982, *P*<0.01). Additionally, all solutions tested produced statistically distinguishable R_ct_ values (*P*<0.05), except for 25 vs. 50% (*P*>0.05).

#### MCF-7 and WGA DNA

The volume of test solution was reduced to 1.8 ml to account for the low quantities of secondary PCR product. A ratio of 1/3 was selected first as this was the ratio utilised by Koo et al. [35]. Further dilutions of 1/18, 1/36, and 1/180 were also tested to determine the limit of detection for the experimental technique (Figure 2E). It was established that only 10 μl of secondary PCR product in 1.8 ml of 1× PBS (1/180) was required to detect the presence of MCF-7 and WGA DNA statistically (*P*<0.05). However, it was not possible to statistically distinguish the EN1 gene promoter amplicon from MCF-7 and WGA DNA until 100 μl of secondary PCR product was used in 1.8 ml of 1× PBS (1/18). This was also the ratio where the greatest difference in R_ct_ between MCF-7 and WGA DNA was observed. Therefore, this ratio was used to test for % methylation in heterogeneous solution (Figure 2F). As with the synthetic oligonucleotides, a negative correlation was observed between % methylation and R_ct_ (r = −0.965, *P*<0.01), however the effect of percentage methylation was more pronounced. Furthermore, the R_ct_ values for 0, 25, 50, 75, and 100% methylated solutions adsorbed on to the 2 mm Au-RDE for 5 min at 2000 rpm were all statistically different from one another when statistically analysed with a one-way ANOVA and Tukey’s post-hoc test (*P*<0.05).

### CV

#### Synthetic oligonucleotides

**Figure 3:**
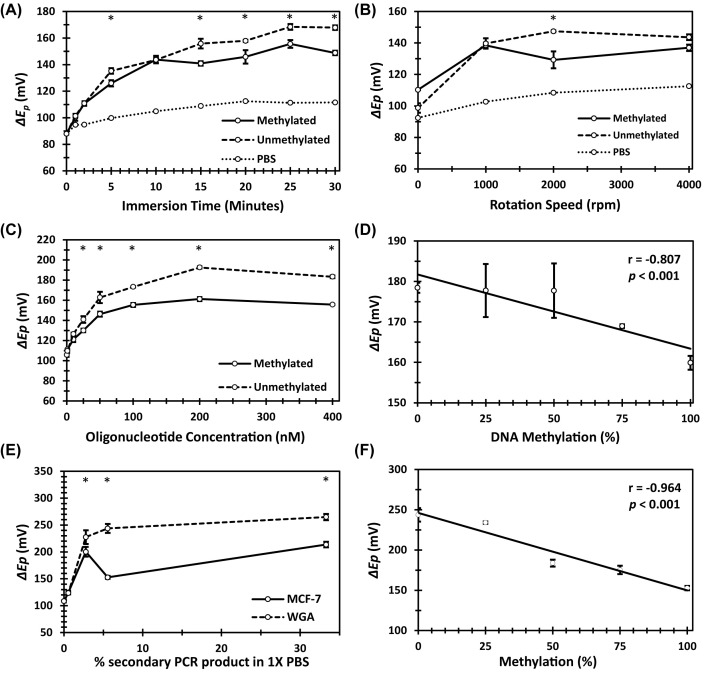
Influence of DNA methylation on *ΔEp* *ΔEp* determined through analysis of cyclic voltammograms for a 2-mm Au-RDE in 2.5 mM ferrocyanide/2.5 mM ferricyanide/1× PBS after adsorption of ssDNA. Optimisation of (**A**) adsorption time of 50 nM ssDNA at a rotation speed of 2000 rpm, (**B**) rotation speed of Au-RDE for adsorption of 50 nM ssDNA for 5 min, and (**C**) oligonucleotide concentration adsorbed at 2000 rpm for 5 min. (**D**) Depicts the correlation between % methylation of heterogeneous samples of 200 nM synthetic oligonucleotides adsorbed on to the Au-RDE for 5 min at 200 rpm, and *ΔEp*. Pearson’s linear correlation coefficient r, and *P* are displayed on plot. (**E**) Optimisation of amount of secondary PCR product, derived from bisulphite-modified MCF-7 and WGA DNA, in 1.8 ml of 1× PBS, adsorbed on to Au-RDE for 5 min at 2000 rpm. Results correspond to 1/180, 1/36, 1/18, and 1/3 dilutions. (**F**) Depicts the correlation between % methylation of heterogeneous samples of 1/18 MCF-7 (methylated) and WGA (unmethylated) DNA adsorbed on to Au-RDE for 5 min at 2000 rpm, and *ΔEp*. Pearson’s linear correlation coefficient r, and *P* are displayed on plot. * indicates *P*<0.05 between methylated and unmethylated DNA in a one-way ANOVA statistical analysis.

Increased adsorption time resulted in an increase in Δ*E_p_* for 50 nM methylated DNA, unmethylated DNA, and 1× PBS (Figure 3A). Cyclic voltammograms and data can be found in Appendix A. Δ*E_p_* or 50 nM methylated and unmethylated DNA was statistically distinguishable at adsorption times of 5, 15, 20, 25, and 30 min, at a rotation speed of 2000 rpm (*P*<0.05). Increased rotation speed resulted in a general elevation in Δ*E_p_* (Figure 3B). As with the results from the analysis of R_ct_, a statistically significant difference was only observed for Δ*E_p_* at 2000 rpm for 50 nM methylated and unmethylated DNA solutions adsorbed on to the 2 mm Au-RDE for 5 min (*P*<0.05). Δ*E_p_* also rose as DNA concentration increased (Figure 3C). The limit of detection was identified as 10 nM for methylated and 25 nM for unmethylated DNA (*P*<0.05). However, DNA solutions were not statistically distinguishable from one another until >25 nM, and again, the greatest difference between DNA samples was observed at 200 nM. As outlined in Figure 3D, Δ*E_p_* was negatively correlated with % methylation in a heterogeneous solution of 200 nM synthetic DNA (r = −0.807, *P*<0.01). CV was a less effective tool for distinguishing % methylation in a heterogeneous sample, as Δ*E_p_* for only 0, 25, and 50% solutions were statistically different against 100% methylation (*P*<0.05), while all other comparisons did not reach statistical significance.

#### MCF-7 and WGA DNA

Δ*E_p_* generally increased as the amount of secondary PCR product in 1× PBS increased (Figure 3E). The limit of detection was established as >50 μl of secondary PCR product in 1.8 ml of 1× PBS (1/36, *P*<0.05). Furthermore, at this level, MCF-7 and WGA amplicons were statistically distinguishable (*P*<0.05). However, the greatest difference between Δ*E_p_* values for MCF-7 and WGA DNA was at 100 μl secondary PCR product used in 1× PBS (1/18). Therefore, this ratio was used to examine % methylation (Figure 3F). A negative correlation between % methylation in heterogeneous solution, and Δ*E_p_* was observed (r = −0.964, *P*<0.01). Again, the effect of % methylation on Δ*E_p_* was more pronounced for MCF-7 and WGA DNA when compared with synthetic oligonucleotides. Δ*E_p_* was less effective at differentiating % methylation for heterogeneous solutions of MCF-7 and WGA DNA, than R_ct_, as Δ*E_p_* for 0% vs. 25 and 50% vs. 75% were not statistically distinguishable (*P*>0.05).

### DPV

#### Synthetic oligonucleotides

**Figure 4:**
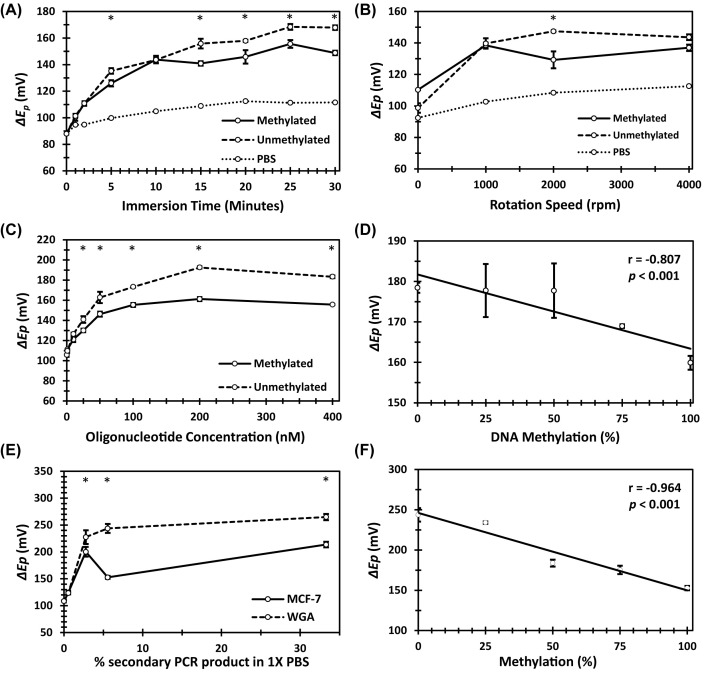
Influence of DNA methylation on *i_pa_* *i_pa_* determined through analysis of differential pulse voltammograms for a 2-mm Au-RDE in 2.5 mM ferrocyanide/2.5 mM ferricyanide/1× PBS after adsorption of ssDNA. Optimisation of (**A**) adsorption time of 50 nM ssDNA at a rotation speed of 2000 rpm, (**B**) rotation speed of Au-RDE for adsorption of 50 nM ssDNA for 5 min, and (**C**) oligonucleotide concentration adsorbed at 2000 rpm for 5 min. (**D**) Depicts the correlation between % methylation of heterogeneous samples of 200 nM synthetic oligonucleotides adsorbed on to the Au-RDE for 5 min at 200 rpm, and *i_pa_*. Pearson’s linear correlation coefficient r, and *P* are displayed on plot. (**E**) Optimisation of amount of secondary PCR product, derived from bisulphite-treated MCF-7 and WGA DNA, in 1.8 ml of 1× PBS, adsorbed on to Au-RDE for 5 min at 2000 rpm. Results correspond to 1/180, 1/36, 1/18, and 1/3 dilutions. (**F**) Depicts the correlation between % methylation of heterogeneous samples of 1/18 MCF-7 (methylated) and WGA (unmethylated) DNA adsorbed on to Au-RDE for 5 min at 2000 rpm, and *i_pa_*. Pearson’s linear correlation coefficient r, and *P* are displayed on plot. * indicates *P*<0.05 between methylated and unmethylated DNA in a one-way ANOVA statistical analysis.

*i_pa_* declined as adsorption time increased. The richer adenine content of the unmethylated DNA sample resulted in a lower *i_pa_* than its methylated counterpart, while the adsorption of 1× PBS on to the 2-mm Au-RDE led to a substantially greater *i_pa_* (Figure 4A). Further to this, *i_pa_* was greatest when no immersion occurred. This can be observed in the differential pulse votammograms and data in the supplementary results file. A statistically significant difference was observed between the *i_pa_* gained for 50 nM methylated and unmethylated DNA adsorbed on to the 2 mm Au-RDE for 5, 20, and 30 min (*P*<0.05). An adsorption time of 5 min was selected to conduct further experimentation at, as it was the minimum time required for a statistical difference to be observed between 50 nM methylated and unmethylated DNA samples (Δ*E_p_* and *i_pa_*). Next, rotation speed was optimised (Figure 4B), where it was established that a statistical difference was only observed at a rotation speed of 2000 rpm (*P*<0.05). Following this, concentration was examined (Figure 4C). As the concentration of both methylated and unmethylated DNA increased, there was a subsequent reduction in *i_pa_*. Interestingly, the limit of detection for methylated DNA was 1 nM, although 10 nM of unmethylated DNA was required to produce a statistically significant difference from 0 nM (*P*<0.05). However, >50 nM was required to differentiate between methylated and unmethylated DNA solutions (*P*<0.05). Using the optimised variables of, adsorption of 200 nM DNA for 5 min at 2000 rpm, a strong positive correlation between % methylation and *i_pa_* was observed (r = 0.902, *P*<0.01) (Figure 4D). *i_pa_* was more effective than CV in differentiating methylation status, as only 0% vs. 25 and 50% vs. 75% solutions could not be distinguished, while all other comparisons reached statistical significance (*P*<0.05).

#### MCF-7 and WGA DNA

A decrease in *i_pa_* was typically observed as the amount of secondary PCR product in 1.8 ml of 1× PBS increased (Figure 4E). Remarkably 10 μl of MCF-7 or WGA DNA in 1.8 ml was sufficient to bring about a statistical difference when compared with 1× PBS. However, 50–100 μl (1/36–1/18) of secondary PCR product in 1.8 ml of 1× PBS was required to differentiate the two amplicons using DPV (*P*<0.05). The greatest difference was observed for 1/18 solutions, where *i_pa_* for MCF-7 DNA was 21.94 ± 0.53 μA compared with 13.90 ± 0.18 μA for WGA DNA. Using this concentration, a strong positive correlation between *i_pa_* and % methylation in a heterogeneous solution was observed (r = 0.856, *P*<0.01), and again the effect was more pronounced (Figure 4F). For instance, when using MCF-7 and WGA DNA, *i_pa_* ranged between 13.90 ± 0.18 and 21.94 ± 0.53 for 0–100% methylation, whereas using synthetic oligonucleotides produced results ranging from 18.34 ± 0.58 to 20.89 ± 0.30. Despite this increase in range, 0 vs. 25%, 0 vs. 50%, and 25 vs. 50% were not statistically different when analysed using a one-way ANOVA with a Tukey’s post-hoc test (*P*>0.05).

## Discussion

The world population is ageing. With an ageing population, there comes a commensurate increase in the prevalence of age-related diseases. Age-related diseases such as CVD and cancer compromise human healthspan and place a significant burden on the wellbeing of older people. It is imperative biomarkers are identified which are capable of predicting age-related diseases. It is conceivable that promoter-specific age-related gene methylation changes are capable of predicting the onset of age-related diseases; while electrochemical techniques provide the necessary foundations for translating this approach into a diagnostic tool. In this work, we have demonstrated that this simple electrochemical procedure can detect DNA methylation in both homogeneous and heterogeneous samples of synthetic ssDNA and breast cancer cell line MCF-7 DNA. Specifically, analysis of 30 base synthetic DNA, designed to represent methylated and unmethylated bisulphite modified and asymmetrically amplified section of the EN1 gene promoter revealed DNA methylation could be detected by EIS, CV, and DPV. Greater R_ct_ (EIS) and ∆E_p_ (CV), and lower *i_pa_* (DPV) were observed for unmethylated samples, demonstrating its higher affinity for the Au-RDE, and thus greater passivation of the Au-RDE, than its methylated counterpart. R_ct_ (EIS) was the most effective electrochemical parameter (method) for the detection of DNA methylation in heterogeneous solutions of synthetic DNA, when the optimum procedure (200 nM DNA for 5 min at 2000 rpm) was employed; followed by *i_pa_* (DPV), and ∆E_p_ (CV). Similarly, Sina et al. (2014) reported that 200 nM was the optimum DNA concentration for creating greatest current difference between samples [36]. Although Koo et al. (2014) reported that a concentration of 50 nM was optimum, this may be because a 53-base sequence containing eight CpG sites was used, in comparison with the 30 base sequence containing six CpG sites utilised in this work [35]. Another difference is that the technique outlined in this work resulted in a 50% reduction in adsorption time, compared with that reported by both Sina et al. (2014) and Koo et al. (2014) [35, 36]. This could be explained by the use of a rotating gold electrode in comparison with a non-motile electrode. While one-time use Au-SPE, as used by Koo et al. (2014), have benefits such as a low cost, ability to change their design and are disposable, their inability to rotate could lead to reduced adsorption. Additionally, it is important to consider that although the Au-RDE requires polishing between tests, while the Au-SPE does not require cleaning due to its disposable nature, the polishing procedure outlined in this study took approximately 3 min to complete. Therefore, this electrode processing time totalled 8 min, 20% less time than required for the disposable Au-SPR. However, it is important to note that this does not include the sample processing time, which incorporates DNA extraction, bisulphite treatment, and asymmetric PCR.

This work provided an insight into the potential of this technique for determining DNA methylation in human-derived samples. For this, the procedure was optimised to 1/18 secondary PCR product in 1× PBS for MFC-7 and WGA DNA. These results again indicated R_ct_ was superior at detecting DNA methylation in heterogeneous samples compared with ∆E_p_, and *i_pa_*. It is vital the sensor is able to detect % methylation in heterogeneous samples, as cancerous masses often exhibit intratumour heterogeneity [42]. It is also important to consider the implications that a technique such as this, may have in detection and monitoring of tumours through a non-invasive blood test, rather than through the more invasive traditional biopsy. Tumour DNA is often released into circulation as cell-free fragments of DNA, and this circulating tumour DNA (ctDNA) has been suggested as a potential biomarker for cancer [43]. Previously ctDNA has been detected in >75% of patients with metastatic cancers, including, but not limited to, bladder, ovarian, breast, colorectal, and hepatocellular cancer [44]. Additionally, a correlation between the number of cancer patients with detectable levels of ctDNA and cancer stage was reported; ctDNA was detected in 47% of patients with stage 1 cancer, 55% of patients with stage 2 cancer, 69% of patients with stage 3 cancer, and 82% of patients with stage 4 cancer. Furthermore, the amount of detectable ctDNA was positively correlated with grade, and negatively correlated with 2-year survival rate [44]. Therefore, this technology could be used to indicate disease severity and prognosis, in addition to determining the presence of the disease.

The low levels of DNA required by the technique outlined in the present paper could be a factor in deciding which method to use to detect DNA methylation. This is because many techniques require larger concentrations of DNA, which could limit their applicability in some instances. For example, ELISA-based kits require >100 ng DNA, while the DNA restriction digest based technique LUMA requires 250–500 ng DNA [45]. Based on previous work which has reported circulating DNA in cancer patients to be on average 219 ng/ml (range 10–1200 ng/ml) while control individuals exhibited <2 ng/ml [46], the developed sensor should be sensitive enough to detect the presence of cancer given the collection of a 5-ml blood sample. However, it is important to recognise the importance of the investigations which used methylation-specific PCR to determine ctDNA methylation, as a biosensor for lung cancer [47]. Results show more than or equal to one out of five genes analysed exhibited aberrant methylation in 77% of lung biopsies from patients, whereas the same result was only observed in 49.5% of samples derived from serum [47]. Similar findings were reported by in a further study which found only 44% of serum samples from cancer patients exhibited hypermethylation of the CDKN2A gene promoter. Therefore, it is important to consider that these so-called liquid biopsies may have reduced sensitivities than traditional biopsies.

In this work, electrochemical techniques were used to detect methylation in breast cancer cells, however it is vital to realise that this technology could be applied to alterative gene promoters for the detection of cancer, such as BRCA1 for breast cancer [48]. Furthermore, it is important to recognise the potential of this technology if it were applied to commonly methylated gene promoters from a larger scope of age-related disease; such as the INS gene promoter in type 2 diabetes mellitus (T2DM) [49], the OPRD1 gene promoter in AD [50], the SLC6A4 gene promoter in obesity [51], or the ABCA1 gene promoter in coronary artery disease (CAD) [52], which have all been shown to be hypermethylated during disease. Moreover, this technology could be used to estimate age itself. This is a logical assumption to make, as Horvaths epigenetic clock describes the methylation trend of 353 CpG sites with age, and has an accuracy of 3.6 years. Within this model, methylation of 193 CpG sites positively correlate with age, and 160 CpG sites negatively correlate with age. Given further optimisation of the electrochemical procedure outlined, it may be possible to use these methods to determine epigenetic age [7].

Moreover, the technique could be refined and improved to investigate the pleiotropic effects methylation can have on genes during different stages of life. There are many developmental genes which are regulated by DNA methylation to allow tissue-specific expression and expression at different times of development, with aberrant methylation resulting in disease [15]. In this work we specifically refer to EN1, a key homoeobox gene, in which aberrant DNA methylation is associated with perturbed gene expression and cancer. For instance, hypermethylation of the EN1 gene in a region far upstream of the promoter was positively correlated with gene expression in invasive breast cancer [53]. Interestingly, reduced levels of methylation within the EN1 gene promoter have been associated with increased expression of EN1 in basal-like breast tumours [54]. Overexpression of EN1 has been observed to behave as a pro-survival transcription factor in basal-like breast cancer [55]. Clinical work has revealed patients with basal-like breast cancer tended to be younger at menarche, have increased parity, and a younger age at first full-term pregnancy [56]. This evidence provides a clear link between DNA methylation and subsequent perturbation of EN1 expression and cancer pathogenesis, with health in females of post-reproductive age. Therefore, the *EN1* gene could be suggested as an example gene which supports the antagonistic pleiotropy theory of ageing. For instance, it is unambiguous the wild-type EN1 gene would be selected for, as mutations result in dramatic malformation or death. However, various studies have reported that changes to the DNA methylation patterns within the gene promoter in later life are associated with cancer. Therefore, the susceptibility of the homeobox gene to aberrant DNA methylation in later life is a trade-off with the beneficial effects of the gene during embryonic development. It is possible therefore that DNA methylation may be a key regulator of the deleterious effects of genes in later life, confer an advantage pre-reproduction.

### Limitations of this work

It is imperative to consider how this technique could be refined and developed in the future. A limitation of the technique used in this investigation is that the samples underwent bisulphite treatment. Recently, progress has been made in the development of electrochemical biosensors [57–60]. These electrochemical bioplatforms determine with high sensitivity and selectivity 5-mC and 5-hmC both at global and gene-specific levels using synthetic oligonucleotides. In this instance, assay times ranged between 45 and 90 min, and crucially are performed without using bisulphite treatment or amplification as a precursor. It is important that advances such as these are embraced, and will lead to the future refinement of this approach.

## Conclusion

Electrochemical techniques are a useful tool for detecting changes in DNA methylation patterns, which can be associated with disease such as cancer. Furthermore, techniques such as these may be able to provide insights into tumour size, grading, and mortality. This work revealed R_ct_ derived from EIS was superior at detecting DNA methylation in heterogeneous samples compared with Δ*E_p_* from CV, and *i_pa_* derived from DPV. This inexpensive and rapid means of testing DNA methylation, was able to detect DNA methylation in heterogeneous samples of both synthetic and human-derived DNA. Detecting percent methylation in a heterogeneous sample is important as often intratumour heterogeneity is often observed within cancerous masses. Furthermore, we describe that it may be possible for future advances in this technology to diagnose cancer through a non-invasive liquid blood test due to the release of ctDNA into blood. We also highlight the potential of this test for the detection of other age-related diseases such as AD, CAD, and T2DM, where aberrant methylation is observed. Moreover the technique could be used to investigate the pleiotropic effects of genes throughout life. This technique will be refined and developed further in the near future by removing the need for samples to undergo bisulphite treatment.

## Supplementary Material

supplementary File

## Abbreviations

Δ*E_p_*: peak to peak separation
*i_pa_*: peak anodic current
AD: Alzheimer’s disease
Au-RDE: gold rotating disk electrode
CAD: coronary artery disease
CpG: cytosine-phosphate-guanine
ctDNA: circulating tumour DNA
CV: cyclic voltammetry
CVD: cardiovascular disease
DPV: differential pulse voltammetry
EIS: electrochemical impedance spectroscopy
*EN1*: engrailed-1
MCF-7: Michigan Cancer Foundation-7
PBS: phosphate-buffered saline
R_ct_: charge transfer resistance
ssDNA: single stranded DNA
T2DM: type 2 diabetes mellitus
WGA: whole genome amplified
5-hmc: 5-hydroxymethylcytosine
5-mc: 5-methylcytosine

## References

[1] Mc Auley M.T., Guimera A.M., Hodgson D., McDonald N., Mooney K.M., Morgan A.E. et al. (2017) Modelling the molecular mechanisms of aging. Biosci. Rep. 37, 1–20 10.1042/BSR20160177 28096317 PMC5322748

[2] Morgan A.E., Davies T.J. and Mc Auley M.T. (2018) The role of DNA methylation in ageing and cancer. Proc. Nutr. Soc. 77, 412–422 10.1017/S0029665118000150 29708096

[3] Benjamin E.J., Muntner P., Alonso A., Bittencourt M.S., Callaway C.W., Carson A.P. et al. (2019) Heart disease and stroke statistics-2019 update: a report from the American Heart Association. Circulation 139, e56–e528 10.1161/CIR.0000000000000659 30700139

[4] Cinar D. and Tas D. (2015) Cancer in the elderly. North. Clin. Istanb. 2, 73–80 28058345 10.14744/nci.2015.72691 PMC5175057

[5] Mc Auley M.T., Mooney K.M., Angell P.J. and Wilkinson S.J. (2015) Mathematical modelling of metabolic regulation in aging. Metabolites 5, 232–251 10.3390/metabo5020232 25923415 PMC4495371

[6] Lipsky M.S. and King M. (2015) Biological theories of aging. Dis. Mon. 61, 460–466 10.1016/j.disamonth.2015.09.005 26490576

[7] Horvath S. (2013) DNA methylation age of human tissues and cell types. Genome Biol. 14, R115 10.1186/gb-2013-14-10-r115 24138928 PMC4015143

[8] Dhingra R., Kwee L.C., Diaz-Sanchez D., Devlin R.B., Cascio W., Hauser E.R. et al. (2019) Evaluating DNA methylation age on the Illumina MethylationEPIC Bead Chip. PLoS ONE 14, e0207834 10.1371/journal.pone.0207834 31002714 PMC6474589

[9] Horvath S. and Raj K. (2018) DNA methylation-based biomarkers and the epigenetic clock theory of ageing. Nat. Rev. Genet. 19, 371–384 10.1038/s41576-018-0004-3 29643443

[10] Jones M.J., Goodman S.J. and Kobor M.S. (2015) DNA methylation and healthy human aging. Aging Cell 14, 924–932 10.1111/acel.12349 25913071 PMC4693469

[11] Zagkos L., Auley M.M., Roberts J. and Kavallaris N.I. (2019) Mathematical models of DNA methylation dynamics: Implications for health and ageing. J. Theor. Biol. 462, 184–193 10.1016/j.jtbi.2018.11.006 30447224

[12] Larson K., Zagkos L., Mc Auley M., Roberts J., Kavallaris N.I. and Matzavinos A. (2019) Data-driven selection and parameter estimation for DNA methylation mathematical models. J. Theor. Biol. 467, 87–99 10.1016/j.jtbi.2019.01.012 30633883

[13] Barciszewska M.Z., Barciszewska A.M. and Rattan S.I.S. (2007) TLC-based detection of methylated cytosine: application to aging epigenetics. Biogerontology 8, 673–678 10.1007/s10522-007-9109-3 17891469

[14] Xiao F.H., Wang H.T. and Kong Q.P. (2019) Dynamic DNA methylation during aging: a “Prophet” of age-related outcomes. Front. Genet. 10, 107 10.3389/fgene.2019.00107 30833961 PMC6387955

[15] Baribault C., Ehrlich K.C., Ponnaluri V.K.C., Pradhan S., Lacey M. and Ehrlich M. (2018) Developmentally linked human DNA hypermethylation is associated with down-modulation, repression, and upregulation of transcription. Epigenetics 13, 275–289 10.1080/15592294.2018.1445900 29498561 PMC5997157

[16] An N., Yang X., Cheng S., Wang G. and Zhang K. (2015) Developmental genes significantly afflicted by aberrant promoter methylation and somatic mutation predict overall survival of late-stage colorectal cancer. Sci. Rep. 5, 18616 10.1038/srep18616 26691761 PMC4686889

[17] Hamilton W.D. (1966) The moulding of senescence by natural selection. J. Theor. Biol. 12, 12–45 10.1016/0022-5193(66)90184-6 6015424

[18] Medawar P.B. (1952) An Unsolved Problem of Biology H.K. Lewis, London

[19] Williams G.C. (1957) Pleiotropy, natural selection, and the evolution of senescence. Evolution 11, 398–411 10.1111/j.1558-5646.1957.tb02911.x

[20] Kirkwood T.B. (1977) Evolution of ageing. Nature 270, 301–304 10.1038/270301a0 593350

[21] Huettl R.-E., Luxenhofer G., Bianchi E., Haupt C., Joshi R., Prochiantz A. et al. (2015) Engrailed 1 mediates correct formation of limb innervation through two distinct mechanisms. PLoS ONE 10, e0118505 10.1371/journal.pone.0118505 25710467 PMC4340014

[22] Wilson S.L., Kalinovsky A., Orvis G.D. and Joyner A.L. (2011) Spatially restricted and developmentally dynamic expression of engrailed genes in multiple cerebellar cell types. Cerebellum 10, 356–372 10.1007/s12311-011-0254-5 21431469 PMC3170510

[23] Zec N., Rowitch D.H., Bitgood M.J. and Kinney H.C. (1997) Expression of the homeobox-containing genes EN1 and EN2 in human fetal midgestational medulla and cerebellum. J. Neuropathol. Exp. Neurol. 56, 236–242 10.1097/00005072-199703000-00002 9056537

[24] Frigola J., Song J., Stirzaker C., Hinshelwood R.A., Peinado M.A. and Clark S.J. (2006) Epigenetic remodeling in colorectal cancer results in coordinate gene suppression across an entire chromosome band. Nat. Genet. 38, 540–549 10.1038/ng1781 16642018

[25] Devaney J., Stirzaker C., Qu W., Song J.Z., Statham A.L., Patterson K.I. et al. (2011) Epigenetic deregulation across chromosome 2q14.2 differentiates normal from prostate cancer and provides a regional panel of novel DNA methylation cancer biomarkers. Cancer Epidemiol. Biomarkers Prev. 20, 148–159 10.1158/1055-9965.EPI-10-0719 21098650

[26] Jiang C.-L., He S.-W., Zhang Y.-D., Duan H.-X., Huang T., Huang Y.-C. et al. (2017) Air pollution and DNA methylation alterations in lung cancer: a systematic and comparative study. Oncotarget 8, 1369–1391 10.18632/oncotarget.13622 27901495 PMC5352062

[27] Bell A., Bell D., Weber R.S. and El-Naggar A.K. (2011) CpG island methylation profiling in human salivary gland adenoid cystic carcinoma. Cancer 117, 2898–2909 10.1002/cncr.25818 21692051 PMC3123690

[28] Mayor R., Casadomé L., Azuara D., Moreno V., Clark S.J., Capellà G. et al. (2009) Long-range epigenetic silencing at 2q14.2 affects most human colorectal cancers and may have application as a non-invasive biomarker of disease. Br. J. Cancer 100, 1534–1539 10.1038/sj.bjc.6605045 19384295 PMC2696749

[29] Rekaik H., Blaudin de The F.X., Fuchs J., Massiani-Beaudoin O., Prochiantz A. and Joshi R.L. (2015) Engrailed homeoprotein protects mesencephalic dopaminergic neurons from oxidative stress. Cell Rep. 13, 242–250 10.1016/j.celrep.2015.08.076 26411690 PMC5066840

[30] Schumacher A., Kapranov P., Kaminsky Z., Flanagan J., Assadzadeh A., Yau P. et al. (2006) Microarray-based DNA methylation profiling: technology and applications. Nucleic Acids Res. 34, 528–542 10.1093/nar/gkj461 16428248 PMC1345696

[31] Herman J.G., Graff J.R., Myöhänen S., Nelkin B.D. and Baylin S.B. (1996) Methylation-specific PCR: a novel PCR assay for methylation status of CpG islands. Proc. Natl. Acad. Sci. U.S.A. 93, 9821–9826 10.1073/pnas.93.18.9821 8790415 PMC38513

[32] Yáñez-Sedeño P., Agüí L., Campuzano S. and Pingarrón J.M. (2019) What electrochemical biosensors can do for forensic science? Unique features and applications. Biosensors (Basel) 9, 1–30 10.3390/bios9040127 PMC6956127 31671772

[33] Fojta M., Daňhel A., Havran L. and Vyskočil V. (2016) Recent progress in electrochemical sensors and assays for DNA damage and repair. Trends Anal. Chem. 79, 160–167 10.1016/j.trac.2015.11.018

[34] Campuzano S., Barderas R., Pedrero M., Yáñez-Sedeño P. and Pingarrón J. (2020) Electrochemical biosensing to move forward in cancer epigenetics and metastasis: a review. Anal. Chim. Acta 1109, 169–190 10.1016/j.aca.2020.01.047 32252900

[35] Koo K.M., Sina A.A., Carrascosa L.G., Shiddiky M.J. and Trau M. (2014) eMethylsorb: rapid quantification of DNA methylation in cancer cells on screen-printed gold electrodes. Analyst 139, 6178–6184 10.1039/C4AN01641F 25318073

[36] Sina A.A.I., Howell S., Carrascosa L.G., Rauf S., Shiddiky M.J. and Trau M. (2014) eMethylsorb: electrochemical quantification of DNA methylation at CpG resolution using DNA–gold affinity interactions. Chem. Commun. 50, 13153–13156 10.1039/C4CC06732K 25227312

[37] Sina A.A., Carrascosa L.G., Liang Z., Grewal Y.S., Wardiana A., Shiddiky M.J.A. et al. (2018) Epigenetically reprogrammed methylation landscape drives the DNA self-assembly and serves as a universal cancer biomarker. Nat. Commun. 9, 4915 10.1038/s41467-018-07214-w 30514834 PMC6279781

[38] Hayatsu H., Shiraishi M. and Negishi K. (2008) Bisulfite modification for analysis of DNA methylation. Curr. Protoc. Nucleic Acid Chem. Chapter 6:Unit 6 10 10.1002/0471142700.nc0610s33 18551428

[39] Heiat M., Ranjbar R., Latifi A.M., Rasaee M.J. and Farnoosh G. (2017) Essential strategies to optimize asymmetric PCR conditions as a reliable method to generate large amount of ssDNA aptamers. Biotechnol. Appl. Biochem. 64, 541–548 10.1002/bab.1507 27222205

[40] Kimura-Suda H., Petrovykh D.Y., Tarlov M.J. and Whitman L.J. (2003) Base-dependent competitive adsorption of single-stranded DNA on gold. J. Am. Chem. Soc. 125, 9014–9015 10.1021/ja035756n 15369348

[41] Sina A.A., Howell S., Carrascosa L.G., Rauf S., Shiddiky M.J. and Trau M. (2014) eMethylsorb: electrochemical quantification of DNA methylation at CpG resolution using DNA-gold affinity interactions. Chem. Commun. (Camb.) 50, 13153–13156 10.1039/C4CC06732K 25227312

[42] Litovkin K., Van Eynde A., Joniau S., Lerut E., Laenen A., Gevaert T. et al. (2015) DNA methylation-guided prediction of clinical failure in high-risk prostate cancer. PLoS ONE 10, e0130651 10.1371/journal.pone.0130651 26086362 PMC4472347

[43] Warton K. and Samimi G. (2015) Methylation of cell-free circulating DNA in the diagnosis of cancer. Front. Mol. Biosci. 2, 13 10.3389/fmolb.2015.00013 25988180 PMC4428375

[44] Bettegowda C., Sausen M., Leary R.J., Kinde I., Wang Y., Agrawal N. et al. (2014) Detection of circulating tumor DNA in early- and late-stage human malignancies. Sci. Transl. Med. 6, 224ra24 10.1126/scitranslmed.3007094 24553385 PMC4017867

[45] Kurdyukov S. and Bullock M. (2016) DNA methylation analysis: choosing the right method. Biology (Basel) 5, 3 10.3390/biology5010003 PMC4810160 26751487

[46] Jahr S., Hentze H., Englisch S., Hardt D., Fackelmayer F.O., Hesch R.D. et al. (2001) DNA fragments in the blood plasma of cancer patients: quantitations and evidence for their origin from apoptotic and necrotic cells. Cancer Res. 61, 1659–1665 11245480

[47] Fujiwara K., Fujimoto N., Tabata M., Nishii K., Matsuo K., Hotta K. et al. (2005) Identification of epigenetic aberrant promoter methylation in serum DNA is useful for early detection of lung cancer. Clin. Cancer Res. 11, 1219–1225 15709192

[48] Zhang L. and Long X. (2015) Association of BRCA1 promoter methylation with sporadic breast cancers: evidence from 40 studies. Sci. Rep. 5, 17869 10.1038/srep17869 26643130 PMC4672329

[49] Yang B.T., Dayeh T.A., Kirkpatrick C.L., Taneera J., Kumar R., Groop L. et al. (2011) Insulin promoter DNA methylation correlates negatively with insulin gene expression and positively with HbA(1c) levels in human pancreatic islets. Diabetologia 54, 360–367 10.1007/s00125-010-1967-6 21104225 PMC3017313

[50] Ji H., Wang Y., Liu G., Chang L., Chen Z., Zhou D. et al. (2017) Elevated OPRD1 promoter methylation in Alzheimer’s disease patients. PLoS ONE 12, e0172335 10.1371/journal.pone.0172335 28253273 PMC5333823

[51] Zhao J., Goldberg J. and Vaccarino V. (2012) Promoter methylation of serotonin transporter gene is associated with obesity measures: a monozygotic twin study. Int. J. Obes. 37, 140 10.1038/ijo.2012.8 PMC3539149 22290534

[52] Guay S.-P., Légaré C., Houde A.-A., Mathieu P., Bossé Y. and Bouchard L. (2014) Acetylsalicylic acid, aging and coronary artery disease are associated with ABCA1 DNA methylation in men. Clin. Epigenetics 6, 14 10.1186/1868-7083-6-14 25093045 PMC4120725

[53] Rauscher G.H., Kresovich J.K., Poulin M., Yan L., Macias V., Mahmoud A.M. et al. (2015) Exploring DNA methylation changes in promoter, intragenic, and intergenic regions as early and late events in breast cancer formation. BMC Cancer 15, 816 10.1186/s12885-015-1777-9 26510686 PMC4625569

[54] Han Y.J., Boatman S.M., Zhang J., Du X.C., Yeh A.C., Zheng Y. et al. (2018) LncRNA BLAT1 is upregulated in basal-like breast cancer through epigenetic modifications. Sci. Rep. 8, 15572 10.1038/s41598-018-33629-y 30349062 PMC6197278

[55] Beltran A.S., Graves L.M. and Blancafort P. (2013) Novel role of Engrailed 1 as a prosurvival transcription factor in basal-like breast cancer and engineering of interference peptides block its oncogenic function. Oncogene 33, 4767 10.1038/onc.2013.422 24141779 PMC4184217

[56] Millikan R.C., Newman B., Tse C.-K., Moorman P.G., Conway K., Dressler L.G. et al. (2008) Epidemiology of basal-like breast cancer. Breast Cancer Res. Treat. 109, 123–139 10.1007/s10549-007-9632-6 17578664 PMC2443103

[57] Povedano E., Vargas E., Montiel V.R., Torrente-Rodríguez R.M., Pedrero M., Barderas R. et al. (2018) Electrochemical affinity biosensors for fast detection of gene-specific methylations with no need for bisulfite and amplification treatments. Sci. Rep. 8, 6418 10.1038/s41598-018-24902-1 29686400 PMC5913137

[58] Bhattacharjee R., Moriam S., Nguyen N.T. and Shiddiky M.J.A. (2019) A bisulfite treatment and PCR-free global DNA methylation detection method using electrochemical enzymatic signal engagement. Biosens. Bioelectron. 126, 102–107 10.1016/j.bios.2018.10.020 30396016

[59] Povedano E., Ruiz-Valdepeñas Montiel V.C., Gamella M., Pedrero M., Barderas R., Peláez-García A. et al. (2020) Amperometric bioplatforms to detect regional DNA methylation with single-base sensitivity. Anal. Chem. 92, 5604–5612 10.1021/acs.analchem.0c00628 32073832

[60] Povedano E., Montiel V.R.-V., Valverde A., Navarro-Villoslada F., Yáñez-Sedeño P., Pedrero M. et al. (2018) Versatile electroanalytical bioplatforms for simultaneous determination of cancer-related DNA 5-methyl-and 5-hydroxymethyl-cytosines at global and gene-specific levels in human serum and tissues. ACS Sensors 4, 227–234 10.1021/acssensors.8b01339 30499292

