# Supplementary material for: Electrochemically detecting DNA methylation in the EN1 gene promoter: implications for understanding ageing and disease
Source: Biosci Rep. 2020 Nov 17;40(11):BSR20202571. doi: 10.1042/BSR20202571 (PMC7670582; doi:10.1042/BSR20202571)
Supplement: supplementary File [file BSR-2020-2571_supp.pdf]

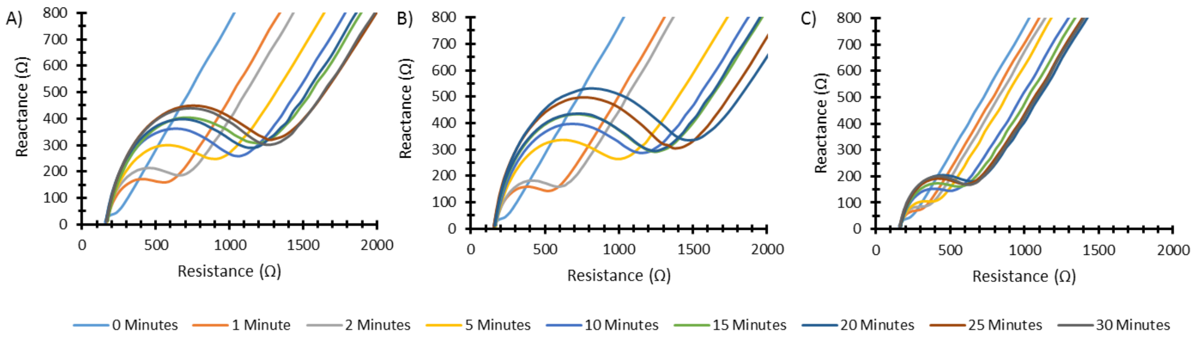

Figure S1 Effect of DNA adsorption time on Nyquist plots.
Nyquist plots for a 2mm Au-RDE in 2.5mM ferrocyanide/2.5mM ferricyanide/1X PBS after rotation assisted adsorption at 2000rpm for a range of times in A) 50nM methylated DNA, B) 50nM unmethylated DNA, C) 1X PBS. Mean shown (n=3, PBS n=1).

Table S1 Effect of DNA adsorption time on R_{ct}.
R_{ct} (determined from Z-fit analysis of Nyquist plots) for a 2mm Au-RDE in 2.5mM ferrocyanide/2.5mM ferricyanide/1X PBS after rotation assisted adsorption at 2000rpm for a range of times in 50nM methylated and unmethylated DNA, and 1X PBS. Mean ±SD (n=3, PBS n = 1). *Statistical difference between values for methylated and unmethylated DNA, when tested for using

Time (Mins)	R_{ct} (Ω)									
	No Immersion			Methylated			Unmethylated			PBS
	Mean	SD	RSD (%)	Mean	SD	RSD (%)	Mean	SD	RSD (%)	
0	76.41	2.59	3.39							
1				263.17	12.79	4.86	323.30	10.67	3.30	140.70
2				335.97	24.06	7.16	395.10	17.29	4.38	178.90
5				707.57	10.62	1.50	795.23	11.62	1.46	228.00
10				844.97	18.15	2.15	937.53	10.25	1.09	343.90
15				967.27	82.51	8.53	1019.87	57.02	5.59	391.40
20				927.57	31.41	3.39	1014.27	65.37	6.45	466.00
25				1062.33	37.00	3.48	1145.33	25.15	2.20	436.30
30				1039.03*	62.48	6.01	1233.33*	67.25	5.45	443.60

a one-way ANOVA (p<0.05).

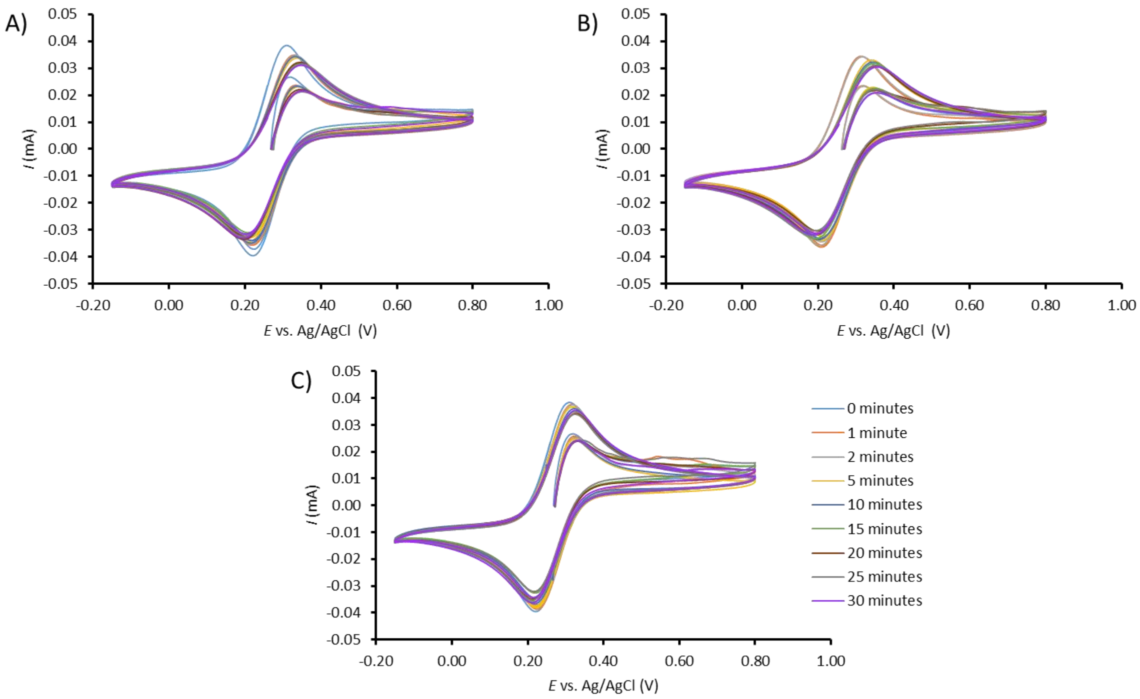

Figure S2 Effect of DNA adsorption time on cyclic voltammograms.
CV conducted for 2mm Au-RDE in 2.5mM ferrocyanide/2.5mM ferricyanide/1X PBS after rotation assisted adsorption at 2000rpm for a range of times in A) 50nM methylated DNA, B) 50nM unmethylated DNA and C) 1X PBS, at a scan speed of 200mV/s. Mean shown (n=3, PBS n=1).

Table S2 Effect of DNA adsorption time on ΔE_p.

Time (Mins)	ΔE_p (mV)									
	No immersion			Methylated			Unmethylated			PBS
	Mean	SD	RSD (%)	Mean	SD	RSD (%)	Mean	SD	RSD (%)	
0	87.9	0.5	0.57							
1				100.3	2.6	2.61	101.3	0.60	0.55	94.6
2				110.7	1.7	1.51	110.8	1.60	1.48	94.9
5				126.0*	2.3	1.83	135.2*	2.10	1.57	99.7
10				143.8	2.8	1.97	143.6	2.00	1.38	104.9
15				140.9*	1.7	1.18	155.8*	3.70	2.36	108.8
20				145.8*	5.0	3.44	157.9*	0.80	0.49	112.5

25				155.6*	2.9	1.86	168.4*	2.20	1.29	111.2
30				148.8*	1.7	1.15	167.9*	1.70	1.04	111.6

ΔE_p (mV) for CV conducted for 2mm Au-RDE in 2.5mM ferrocyanide/2.5mM ferricyanide/1X PBS after rotation assisted adsorption at 2000rpm for a range of times in 50nM methylated DNA, 50nM unmethylated DNA and 1X PBS, at a scan speed of 200mV/s. Mean ±SD (n=3, PBS n=1). *Statistical difference between values for methylated and unmethylated DNA, when tested for using a one-way ANOVA (p<0.05).

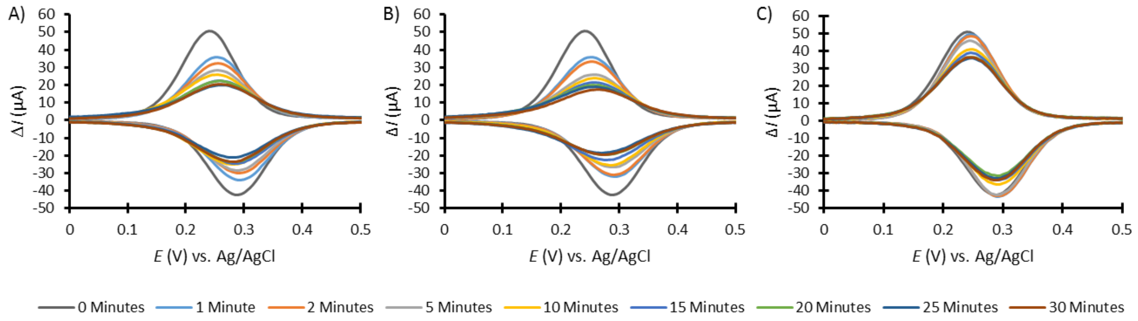

Figure S3 Effect of DNA adsorption time on DPV.
DPV signals for 2mm Au-RDE in 2.5mM ferrocyanide/2.5mM ferricyanide/1X PBS after rotation assisted adsorption at 2000rpm for a range of times in A) 50nM methylated DNA, B) 50nM unmethylated DNA and C) 1X PBS. Mean (n=3, PBS n=1).

Table S3 Effect of DNA adsorption time on peak current.
i_{pa} (µA) obtained with 2mm Au-RDE in 2.5mM ferrocyanide/2.5mM ferricyanide/1X PBS after rotation assisted adsorption at 2000rpm for a range of times in 50nM methylated DNA, 50nM unmethylated DNA and 1X PBS. Mean ±SD (n=3, PBS n=1). *Statistical difference between values for methylated and unmethylated DNA, when tested for using a one-way ANOVA (p<0.05).

Time (Mins)	i_{pa} (µA)									
	No immersion			Methylated			Unmethylated			PBS
	Mean	SD	RSD (%)	Mean	SD	RSD (%)	Mean	SD	RSD (%)	
0	50.82	1.02	2.01							
1				35.66	0.42	1.17	35.67	0.44	1.23	49.55
2				32.18	0.71	2.22	33.40	0.38	1.13	48.15
5				28.65*	0.56	1.95	26.09*	0.34	1.30	45.94
10				25.66	0.42	1.65	23.81	0.12	0.50	40.91

15	22.67	1.01	4.45	21.65	0.29	1.33	38.99
20	22.33*	0.29	1.28	20.18*	1.23	6.09	36.17
25	19.87	0.51	2.56	18.98	0.34	1.78	36.01
30	20.64*	0.61	2.95	17.72*	0.32	1.82	36.13

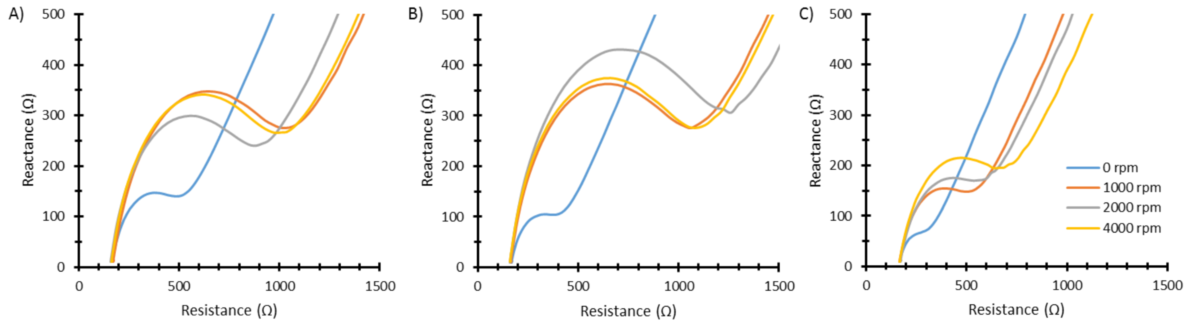

Figure S4 Effect of rotation speed on Nyquist plots.
Nyquist plots for a 2mm Au-RDE in 2.5mM ferrocyanide/2.5mM ferricyanide/1X PBS after rotation assisted adsorption at varying rotation speeds for 5 minutes in A) 50nM methylated DNA, B) 50nM unmethylated DNA, and C) 1X PBS. Mean shown (n=3, 1X PBS n=1).

Table S4 Effect of rotation speed on R_{ct}.
R_{ct} (determined from Z-fit analysis of Nyquist plots) for 2mm Au-RDE in 2.5mM ferrocyanide/2.5mM ferricyanide/1X PBS after rotation assisted adsorption at varying rotation speeds for 5 minutes in 50nM methylated DNA, 50nM unmethylated DNA, and 1X PBS. Mean ±SD (n=3, PBS n=1). *Statistical difference between values for methylated and unmethylated DNA, when tested for using a one-way ANOVA (p<0.05).

Rotation Speed (rpm)	R_{ct} (Ω)						
	Methylated			Unmethylated			PBS
	Mean	SD	RSD (%)	Mean	SD	RSD (%)	
0	326.83	4.87	1.49	231.87	14.95	6.45	140.60
1000	817.67	24.93	3.05	846.87	43.67	5.16	345.00
2000	684.00*	53.21	7.78	1005.43*	32.35	3.22	396.90
4000	792.37	6.29	0.79	868.93	49.16	5.66	502.60

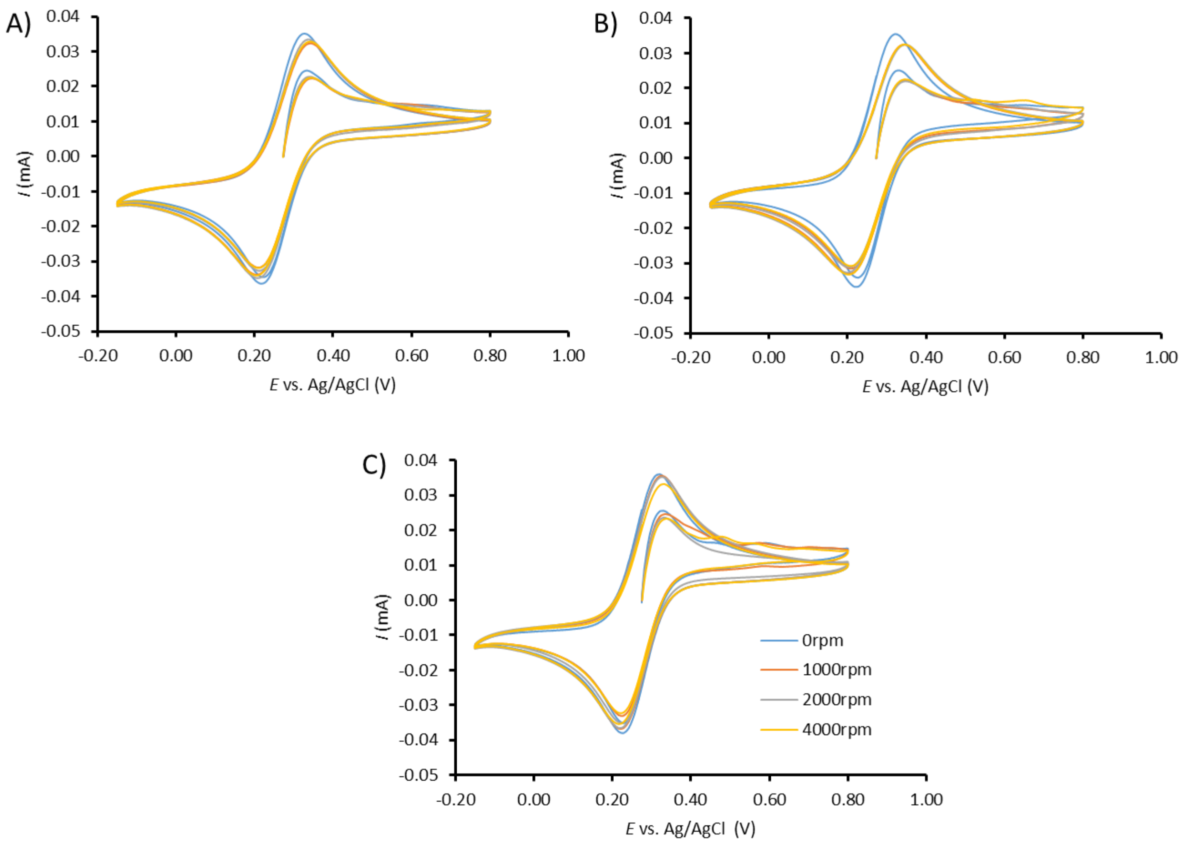

Figure S5 Effect of rotation speed on Cyclic voltammograms.
CV conducted with 2mm Au-RDE in 2.5mM ferrocyanide/2.5mM ferricyanide/1X PBS after rotation assisted adsorption at varying rotation speeds for 5 minutes in A) 50nM methylated DNA, B) 50nM unmethylated DNA and C) 1X PBS, at a scan speed of 200mV/s. Mean ±SD (n=3, PBS n=1).

Table S5 Effect of rotation speed on peak to peak separation.
ΔE_p (mV) for CV conducted with 2mm Au-RDE in 2.5mM ferrocyanide/2.5mM ferricyanide/1X PBS after rotation assisted adsorption at varying rotation speeds for 5 minutes in 50nM methylated DNA, 50nM unmethylated DNA and 1X PBS, at a scan speed of 200mV/s. Mean ±SD (n=3, PBS n=1). *Statistical difference between values for methylated and unmethylated DNA, when tested for using a one-way ANOVA (p<0.05).

Rotation Speed (rpm)	ΔE_p (mV)		
	Methylated	Unmethylated	PBS

	mean	SD	RSD (%)	mean	SD	RSD (%)	
0	110.3	0.1	0.06	98.7	3.0	3.03	92.4
1000	138.5	1.7	1.26	139.7	3.3	2.33	102.7
2000	129.3*	5.4	4.17	147.4*	0.6	0.41	108.4
4000	137.0	2.0	1.46	143.7	1.8	1.28	112.6

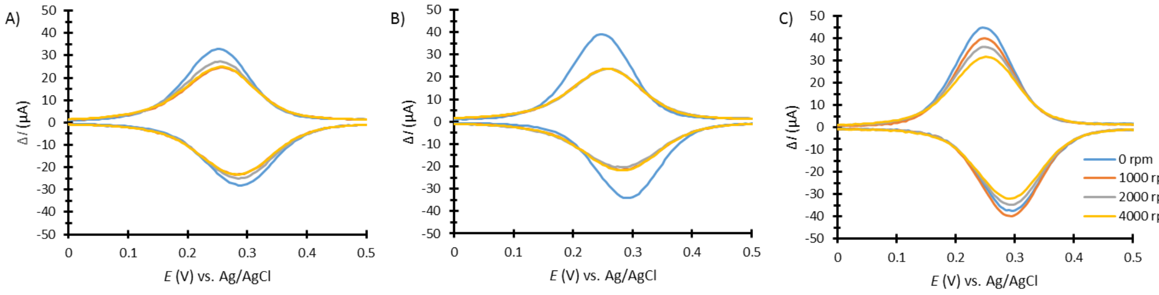

Figure S6 Effect of rotation speed on DPV.
DPV signals for 2mm Au-RDE in 2.5mM ferrocyanide/2.5mM ferricyanide/1X PBS after rotation assisted adsorption at varying rotation speeds for 5 minutes in A) 50nM methylated DNA, B) 50nM unmethylated DNA and C) 1X PBS. Mean (n=3, PBS n=1).

Table S6 Effect of rotation speed on peak current.
i_{pa} (μA) obtained with 2mm Au-RDE in 2.5mM ferrocyanide/2.5mM ferricyanide/1X PBS after rotation assisted adsorption at varying rotation speeds for 5 minutes in 50nM methylated DNA, 50nM unmethylated DNA and 1X PBS. Mean (n=3, PBS n=1). *Statistical difference between values for methylated and unmethylated DNA, when tested for using a one-way ANOVA (p<0.05).

Rotation Speed (rpm)	i_{pa} (μA)						
	Methylated			Unmethylated			PBS
	Mean	SD	RSD (%)	Mean	SD	RSD (%)	
0	32.88	0.33	1.01	39.20	0.57	1.47	44.72
1000	24.58	1.19	4.83	23.64	0.32	1.37	39.93
2000	27.32*	0.75	2.73	23.50*	0.65	2.77	36.19
4000	25.00	0.25	1.02	23.66	1.03	4.33	31.75

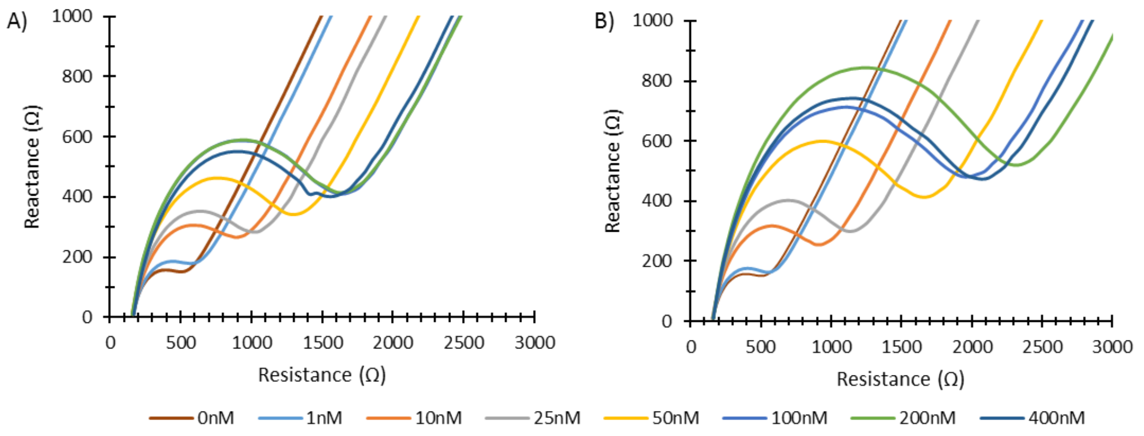

Figure S7 Effect of DNA concentration on impedance.
Nyquist plots for a 2mm Au-RDE in 2.5mM ferrocyanide/2.5mM ferricyanide/1X PBS after rotation assisted adsorption at 2000rpm for 5 minutes in varying concentrations of A) methylated DNA, and B) unmethylated DNA. Mean ±SD (n=3).

Table S7 Effect of DNA concentration on R_{ct}.
R_{ct} (determined from Z-fit analysis of Nyquist plots) for a 2mm Au-RDE in 2.5mM ferrocyanide/2.5mM ferricyanide/1X PBS after rotation assisted adsorption at 2000rpm for 5 minutes in varying concentrations of methylated and unmethylated DNA. Mean ±SD (n=3). *Statistical difference between values for methylated and unmethylated DNA, when tested for using a one-way ANOVA (p<0.05), †statistical difference to value for baseline 1X PBS (p<0.05).

Concentration (nM)	R_{ct} (Ω)								
	PBS			Methylated			Unmethylated		
	Mean	SD	RSD (%)	Mean	SD	RSD (%)	Mean	SD	RSD (%)
0	346.27	31.34	9.05						
1				419.73	16.60	3.95	388.80	9.09	2.34
10				710.73†	15.10	2.12	722.00†	21.20	2.94
25				813.97*†	31.11	3.82	926.17*†	15.51	1.68
50				1078.33*†	35.02	3.25	1403.67*†	38.07	2.71
100				1385.00*†	52.42	3.78	1696.67*†	35.00	2.06
200				1387.00*†	60.23	4.34	1999.67*†	20.53	1.03
400				1309.67*†	16.65	1.27	1770.67*†	17.95	1.01

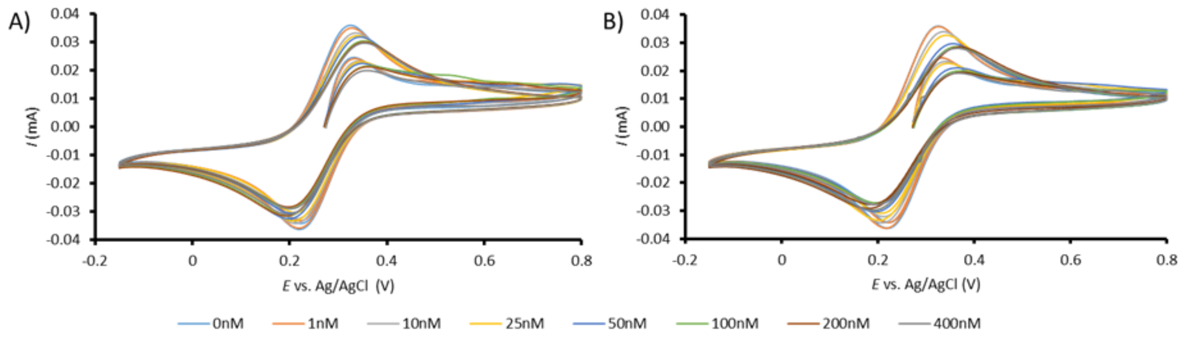

Figure S8 Effect of DNA concentration on cyclic voltammograms.

Cyclic voltammograms for a 2mm Au-RDE in 2.5mM ferrocyanide/2.5mM ferricyanide/1X PBS after rotation assisted adsorption at 2000rpm for 5 minutes in varying concentrations of A) methylated and B) unmethylated DNA, at a scan speed of 200mV/s. Mean ±SD (n=3).

Table S8 Effect of DNA concentration on peak to peak separation.

ΔE_p (mV) for CV conducted with a 2mm Au-RDE in 2.5mM ferrocyanide/2.5mM ferricyanide/1X PBS after rotation assisted adsorption at 2000rpm for 5 minutes in varying concentrations of methylated and unmethylated DNA, at a scan speed of 200mV/s. Mean ±SD (n=3). *Statistical difference between values for methylated and unmethylated DNA, when tested for using a one-way ANOVA

Concentration (nM)	ΔE_p (mV)								
	PBS			Methylated			Unmethylated		
	Mean	SD	RSD (%)	Mean	SD	RSD (%)	Mean	SD	RSD (%)
0	105.8	2.0	1.91						
1				111.1	0.8	0.73	109.7	1.7	1.60
10				121.1†	2.6	2.12	126.4†	1.4	1.07
25				130.0*†	1.7	1.32	140.9*†	3.3	2.33
50				146.3*†	2.4	1.67	162.8*†	5.6	3.44
100				155.4*†	2.0	1.27	173.3*†	0.7	0.38
200				161.2*†	2.0	1.21	192.6*†	1.1	0.55
400				155.7*†	0.3	0.18	183.3*†	1.3	0.70

(p<0.05), †statistical difference to value for baseline 1X PBS (p<0.05).

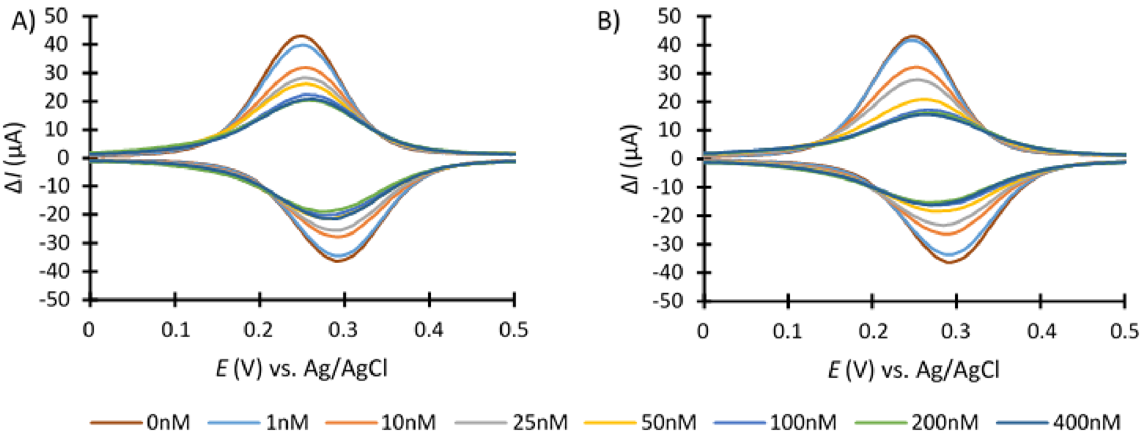

Figure S9 Effect of DNA concentration on DPV.

DPV signals for a 2mm Au-RDE in 2.5mM ferrocyanide/2.5mM ferricyanide/1X PBS after rotation assisted adsorption at 2000rpm for 5 minutes in varying concentrations of A) methylated DNA and B) unmethylated DNA. Mean ±SD (n=3).

Table S9 Effect of DNA concentration on peak anodic current.

i_{pa} (μA) obtained with a 2mm Au-RDE in 2.5mM ferrocyanide/2.5mM ferricyanide/1X PBS after rotation assisted adsorption at 2000rpm for 5 minutes in varying concentrations of methylated and unmethylated DNA. Mean ±SD (n=3). *Statistical difference between values for methylated and unmethylated DNA, when tested for using a one-way ANOVA (p<0.05), †statistical difference to value for baseline 1X PBS (p<0.05).

Concentration (nM)	i_{pa} (μA)								
	PBS			Methylated			Unmethylated		
	mean	SD	RSD (%)	mean	SD	RSD (%)	mean	SD	RSD (%)
0	43.09	1.37	3.17						
1				39.93†	0.56	1.39	41.74	0.53	1.28
10				31.93†	0.56	1.76	32.23†	0.39	1.21
25				28.32†	0.34	1.20	27.85†	0.78	2.81
50				26.23*†	0.35	1.35	20.94*†	0.63	3.01
100				22.43*†	0.28	1.26	17.23*†	0.11	0.65
200				19.92*†	0.17	0.84	16.17*†	0.83	5.13
400				20.86*†	0.16	0.78	15.63*†	0.14	0.89

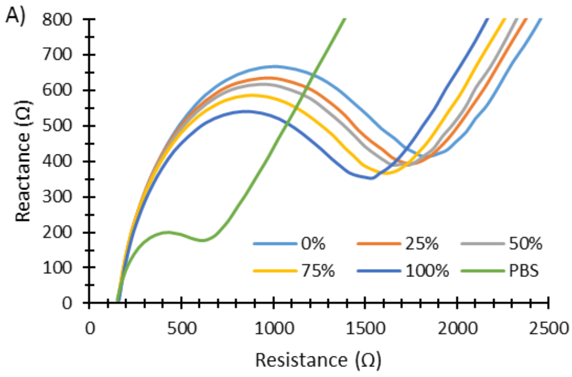

Figure Error! No text of specified style in document.**.10 Effect of % methylation on impedance.** Nyquist plots for a 2mm Au-RDE in 2.5mM ferrocyanide/2.5mM ferricyanide/1X PBS after rotation assisted adsorption at 2000rpm for 5 minutes in varying % methylation solutions (200nM), and 1X PBS (mean, n=3). Mean ±SD (n=3). 1X PBS R_{ct} 454.17 ±37.08 Ω.

Table S10 Effect of % methylation on R_{ct}.
R_{ct} (determined from Z-fit analysis of Nyquist plots) for a 2mm Au-RDE in 2.5mM ferrocyanide/2.5mM ferricyanide/1X PBS after rotation assisted adsorption at 2000rpm for 5 minutes in varying % methylation solutions (200nM) and 1X PBS. Mean ±SD (n=3).

% methylation	R_{ct} (Ω)		
	Mean	SD	RSD (%)
PBS	454.17	37.08	8.16
0	1560.67	25.15	1.61
25	1473.67	6.66	0.45
50	1426.67	21.59	1.51
75	1351.33	27.32	2.02
100	1251.33	12.01	0.96

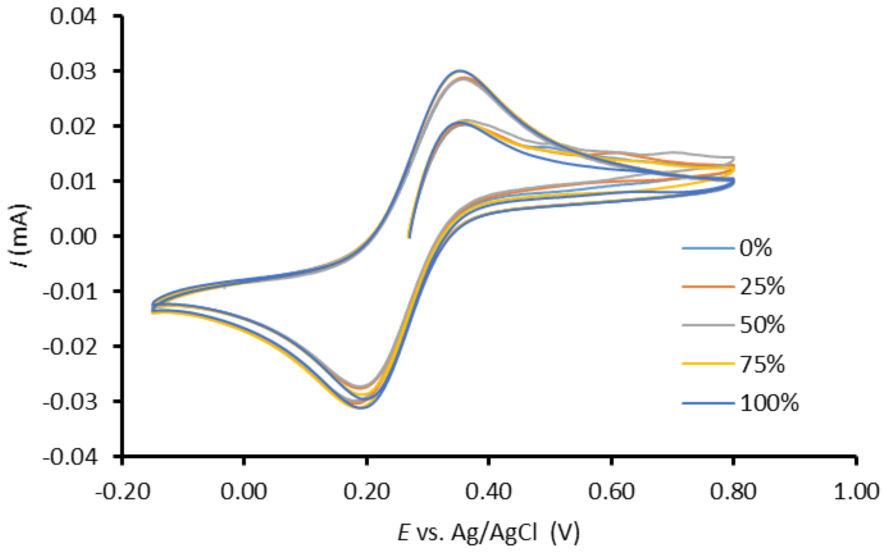

Figure S11 Effect of % methylation on cyclic voltammogram.
CV conducted with a 2mm Au-RDE in 2.5mM ferrocyanide/2.5mM ferricyanide/1X PBS after rotation assisted adsorption at 2000rpm for 5 minutes in varying % methylation solutions (200nM), at a scan speed of 200mV/s. Mean ±SD (n=3).

Table S11 Effect of % methylation on peak to peak separation.
ΔE_p (mV) for CV conducted with a 2mm Au-RDE in 2.5mM ferrocyanide/2.5mM ferricyanide/1X PBS after rotation assisted adsorption at 2000rpm for 5 minutes in varying % methylation solutions (200nM) and 1X PBS, at a scan speed of 200mV/s. Mean ±SD (n=3).

% Methylation	ΔE_p (mV)		
	Mean	SD	RSD (%)
PBS	115.3	8.0	6.93
0	178.5	1.2	0.70
25	177.8	6.6	3.70
50	177.7	6.7	3.79
75	169.0	0.5	0.27
100	159.9	1.7	1.07

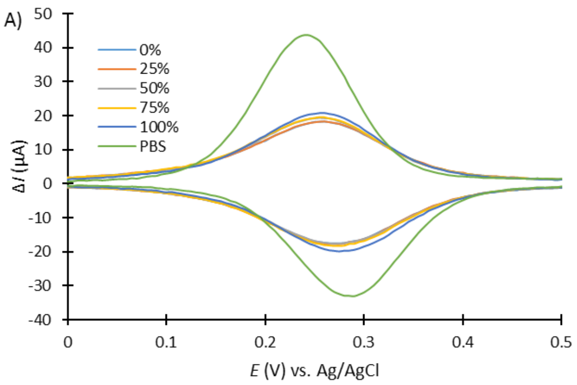

Figure S12 Effect of % methylation on DPV.
DPV signals for 2mm Au-RDE in 2.5mM ferrocyanide/2.5mM ferricyanide/1X PBS after rotation assisted adsorption at 2000rpm for 5 minutes in varying % methylation solutions (200nM) and 1X PBS. Mean ±SD (n=3). PBS i_{pa} 43.72±2.82μA.

Table S12 Effect of % methylation on peak anodic current.
i_{pa} (μA) for 2mm Au-RDE in 2.5mM ferrocyanide/2.5mM ferricyanide/1X PBS after rotation assisted adsorption at 2000rpm for 5 minutes in varying % methylation solutions (200nM). Mean ±SD (n=3).

% Methylation	i_{pa} (μA)		
	Mean	SD	RSD (%)
PBS	43.72	2.82	6.44
0	18.34	0.58	3.16
25	18.38	0.11	0.61
50	19.38	0.12	0.61
75	19.63	0.27	0.61
100	20.89	0.30	1.42

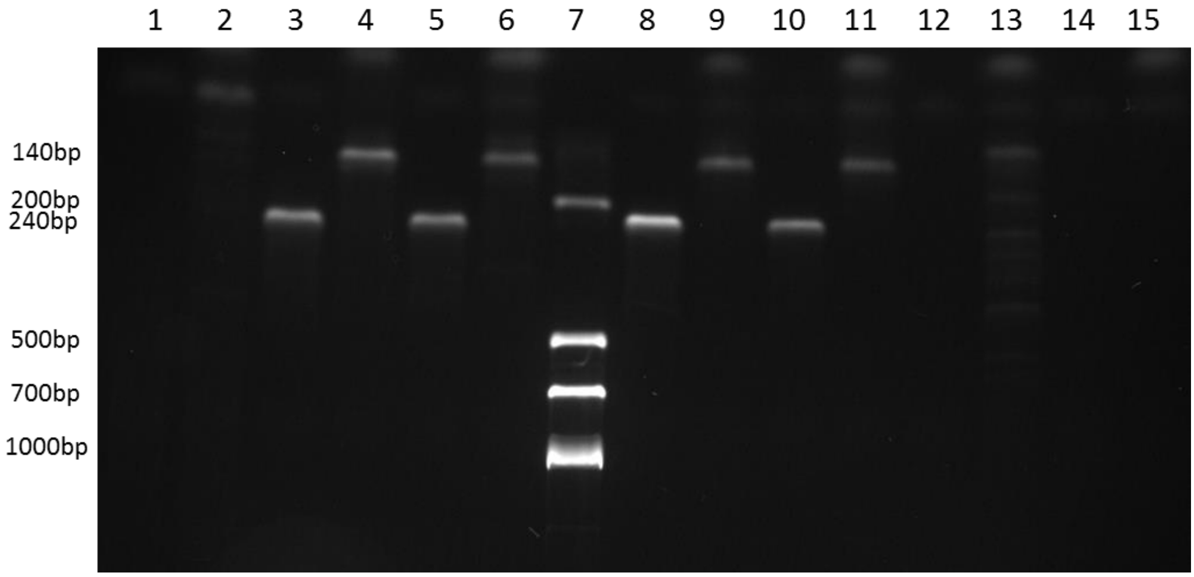

Figure S13 Gel electrophoresis analysis.

Electrophoresis run at 100V for 90 minutes on 2% agarose. Each lane contains: WGA DNA with 1) bisulphite kit primers 2) Koo et al. (2014) primers; bisulphite modified WGA DNA with 3) bisulphite kit primers, 4) Koo et al. (2014) primers; bisulphite modified MCF-7 DNA with 5) bisulphite kit primers, 6) Koo et al. (2014) primers; 7) ladder, standard bands 1000, 700, 500 and 200bp; bisulphite modified control sample with 8) bisulphite kit primers, 9) Koo et al. (2014) primers; pre-treated positive control with 10) bisulphite kit primers, 11) Koo et al. (2014) primers; untreated negative control sample with 12) bisulphite kit primers, 13) Koo et al. (2014) primers; DNA negative sample with 14) bisulphite kit primers, 15) Koo et al. (2014) primers.

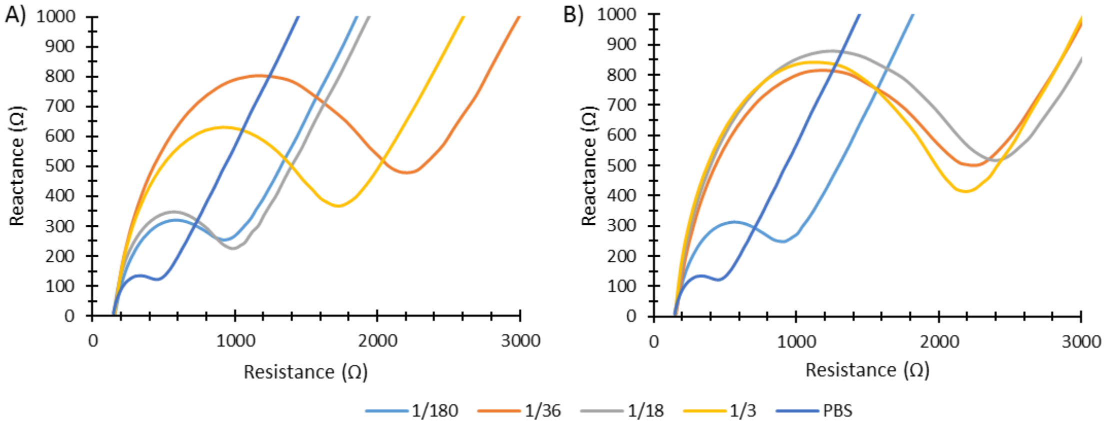

Figure S14 Effect fractional proportion of secondary PCR product in test solution on impedance. Nyquist plots for a 2mm Au-RDE in 2.5mM ferrocyanide/2.5mM ferricyanide/1X PBS after rotation assisted adsorption at 2000rpm for 5 minutes in varying dilutions of amplicons of the EN1 gene promoter from A) MCF-7 DNA and B) WGA DNA. Mean ±SD (n=3).

Table S13 Effect of fractional proportion of secondary PCR product in test solution on R_{ct}. R_{ct} (determined from Z-fit analysis of Nyquist plots) for a 2mm Au-RDE in 2.5mM ferrocyanide/2.5mM ferricyanide/1X PBS after rotation assisted adsorption at 2000rpm for 5 minutes in varying dilutions of amplicons of the EN1 gene promoter from MCF-7 and WGA DNA against 1X PBS. Mean ±SD (n=3). *Statistical difference between values for EN1 amplicons from MCF-7 and WGA DNA, when tested for using a one-way ANOVA ($p<0.05$), †statistical difference to value for baseline 1X PBS ($p<0.05$).

Fraction	R_{ct} (Ω)								
	PBS			MCF-7 DNA			WGA DNA		
	Mean	SD	RSD (%)	Mean	SD	RSD (%)	Mean	SD	RSD (%)
0	296.27	10.30	3.48						
1/180				724.63†	9.58	1.32	698.03†	22.67	3.25
1/36				1901.33†	83.07	4.37	1931.67†	75.08	3.89
1/18				765.17*†	15.01	1.96	2073.67*†	28.54	1.38
1/3				1444.67*†	23.18	1.60	1871.33*†	77.36	4.13

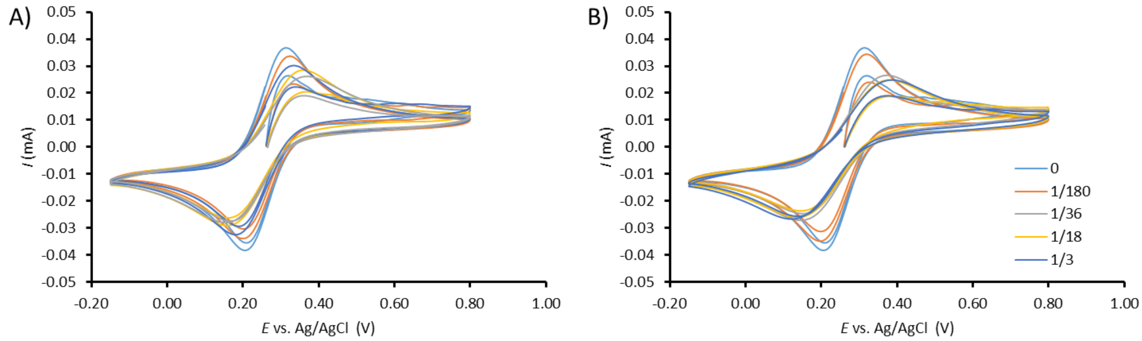

Figure S15 Effect of fractional proportion of secondary PCR product in test solution on cyclic voltammograms.
CV conducted with a 2mm Au-RDE in 2.5mM ferrocyanide/2.5mM ferricyanide/1X PBS after rotation assisted adsorption at 2000rpm for 5 minutes in varying dilutions of amplicons of the EN1 gene promoter from A) MCF-7 and B) WGA DNA, against 1X PBS at a scan speed of 200mV/s. Mean ±SD (n=3).

Table S14 Effect of fractional proportion of secondary PCR product in test solution on peak to peak separation.
ΔE_p (mV) for CV conducted with a 2mm Au-RDE in 2.5mM ferrocyanide/2.5mM ferricyanide/1X PBS after rotation assisted adsorption at 2000rpm for 5 minutes in varying dilutions of amplicons of the EN1 gene promoter from MCF-7 and WGA DNA, against 1X PBS at a scan speed of 200mV/s. Mean ±SD (n=3). *Statistical difference between values for EN1 amplicons from MCF-7 and WGA DNA, when tested for using a one-way ANOVA (p<0.05), †statistical difference to value for baseline 1X PBS

Fraction	ΔE_p (mV)								
	PBS			MCF-7 DNA			WGA DNA		
	Mean	SD	RSD (%)	Mean	SD	RSD (%)	Mean	SD	RSD (%)
0	108.5	2.5	2.32						
1/180				124.3	3.4	2.71	123.6	0.3	0.28
1/36				200.3*†	8.9	4.46	227.6*†	12.6	5.55
1/18				152.7*†	2.8	1.80	243.7*†	8.4	3.46
1/3				213.7*†	5.1	2.40	264.9*†	5.7	2.15

(p<0.05).

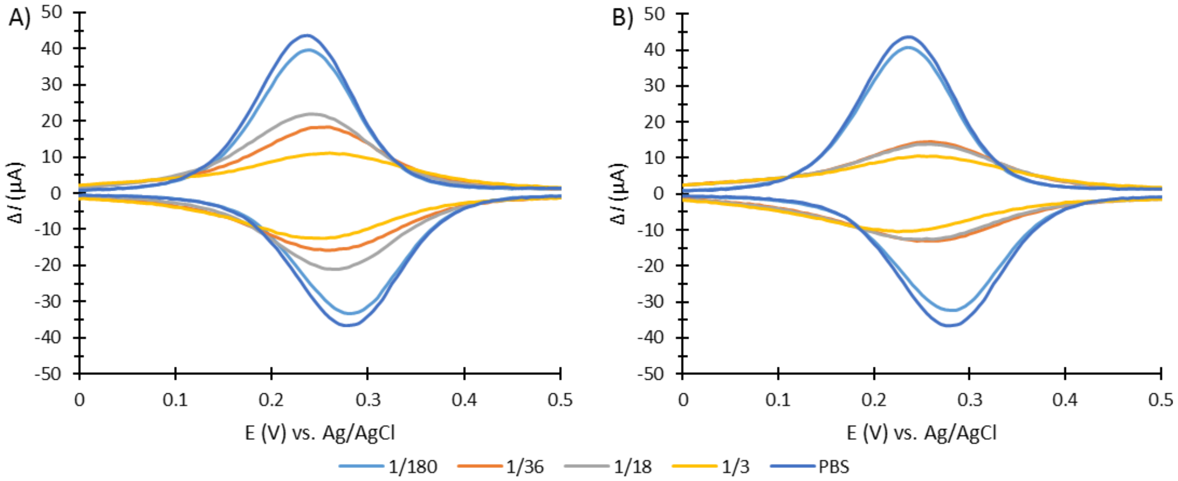

Figure S16 Effect of fractional proportion of secondary PCR product in test solution on DPV.
DPV signals for a 2mm Au-RDE in 2.5mM ferrocyanide/2.5mM ferricyanide/1X PBS after rotation assisted adsorption at 2000rpm for 5 minutes in varying dilutions of amplicons of the EN1 gene promoter from A) MCF-7 DNA and B) WGA DNA. Mean ±SD (n=3).

Table S15 Effect of fractional proportion of secondary PCR product in test solution on peak current.
i_{pa} obtained with 2mm Au-RDE in 2.5mM ferrocyanide/2.5mM ferricyanide/1X PBS after rotation assisted adsorption at 2000rpm for 5 minutes in varying dilutions of amplicons of the EN1 gene promoter from MCF-7 and WGA DNA against 1X PBS. Mean ±SD (n=3). *Statistical difference between values for EN1 amplicons from MCF-7 and WGA DNA, when tested for using a one-way ANOVA (*p*<0.05), †statistical difference to value for baseline 1X PBS (*p*<0.05).

Fraction	i_{pa} (μA)								
	PBS			MCF-7 DNA			WGA DNA		
	mean	SD	RSD (%)	mean	SD	RSD (%)	mean	SD	RSD (%)
0	43.61	0.25	0.58						
1/180				$39.61^{\dagger}$	1.05	2.65	$40.73^{\dagger}$	1.08	2.65
1/36				$18.47^{*\dagger}$	2.17	11.77	$14.66^{*\dagger}$	1.11	7.55
1/18				$21.94^{*\dagger}$	0.53	2.42	$13.90^{*\dagger}$	0.18	1.32
1/3				$11.16^{\dagger}$	0.53	4.74	$10.44^{\dagger}$	0.81	7.78

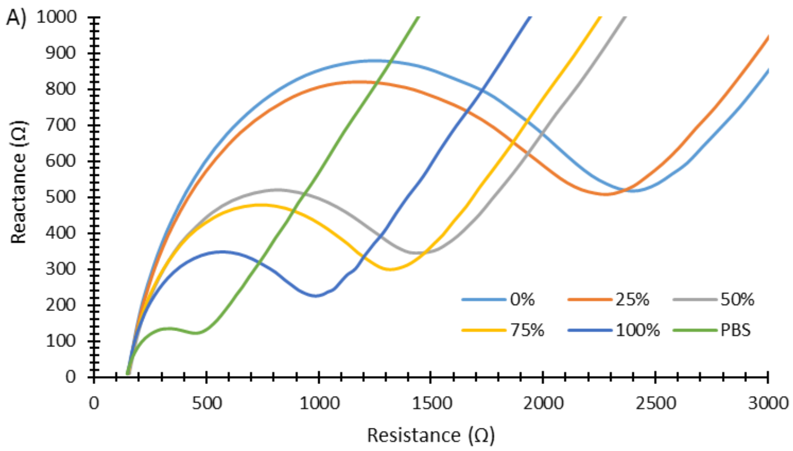

Figure S17 Effect of MCF-7/WGA percentage in test solution on impedance.
Nyquist plot for a 2mm Au-RDE in 2.5mM ferrocyanide/2.5mM ferricyanide/1X PBS after rotation assisted adsorption at 2000rpm for 5 minutes in varying % methylation solutions (mean, n=3). Methylated and unmethylated DNA derived from amplicons of the EN1 gene promoter from MCF-7 and WGA DNA respectively. Secondary PCR product of amplicons total 1/18 in solution. Mean ±SD (n=3).

Table S16 Effect of MCF-7/WGA percentage in test solution on R_{ct}.
R_{ct} (determined from Z-fit analysis of Nyquist plots) for a 2mm Au-RDE in 2.5mM ferrocyanide/2.5mM ferricyanide/1X PBS after rotation assisted adsorption at 2000rpm for 5 minutes in varying % methylation solutions. Methylated and unmethylated DNA derived from amplicons of the EN1 gene promoter from MCF-7 and WGA DNA respectively. Secondary PCR product of amplicons total 1/18 in solution. Mean ±SD (n=3).

% methylation	R_{ct} (Ω)		
	Mean	SD	RSD (%)
PBS	296.27	10.30	3.48
0	2073.67	28.54	1.38
25	1962.00	31.43	1.60
50	1203.67	1.53	0.13
75	1083.00	7.55	0.70
100	765.17	15.01	1.96

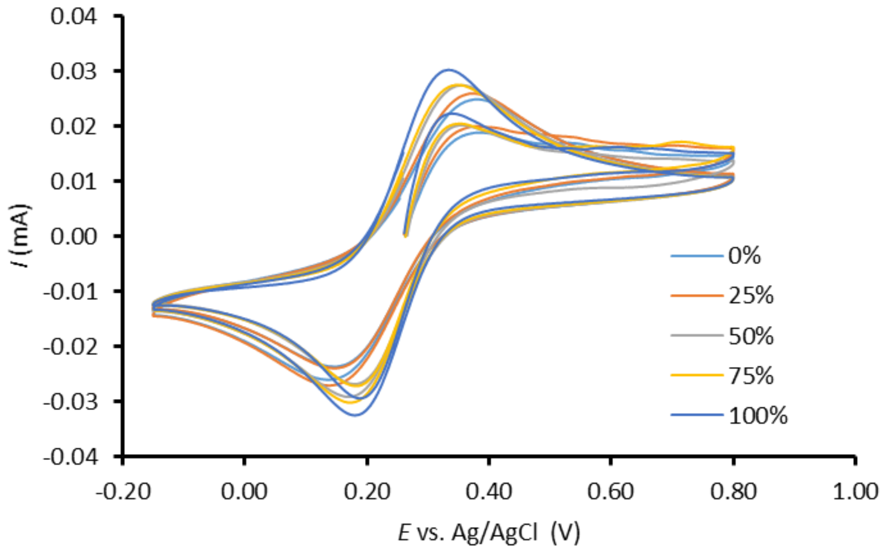

Figure S18 Effect of MCF-7/WGA percentage in test solution on cyclic voltammogram.
CV conducted with a 2mm Au-RDE in 2.5mM ferrocyanide/2.5mM ferricyanide/1X PBS after rotation assisted adsorption at 2000rpm for 5 minutes in varying % methylation solutions at a scan speed of 200mV/s. Methylated and unmethylated DNA derived from amplicons of the EN1 gene promoter from MCF-7 and WGA DNA respectively. Secondary PCR product of amplicons total 1/18 in solution. Mean ±SD (n=3).

Table S17 Effect of MCF-7/WGA percentage in test solution on peak to peak separation.
ΔE_p (mV) for CV conducted with a 2mm Au-RDE in 2.5mM ferrocyanide/2.5mM ferricyanide/1X PBS after rotation assisted adsorption at 2000rpm for 5 minutes in varying % methylation solutions at a scan speed of 200mV/s. Methylated and unmethylated DNA derived from amplicons of the EN1 gene promoter from MCF-7 and WGA DNA respectively. Secondary PCR product of amplicons total 1/18 in solution. Mean ±SD (n=3).

% Methylation	ΔE_p (mV)		
	Mean	SD	RSD (%)
PBS	108.5	2.5	2.32
0	243.7	8.4	3.46
25	234.0	1.5	0.62
50	183.7	4.2	2.30
75	175.3	5.2	2.99
100	152.7	2.8	1.80

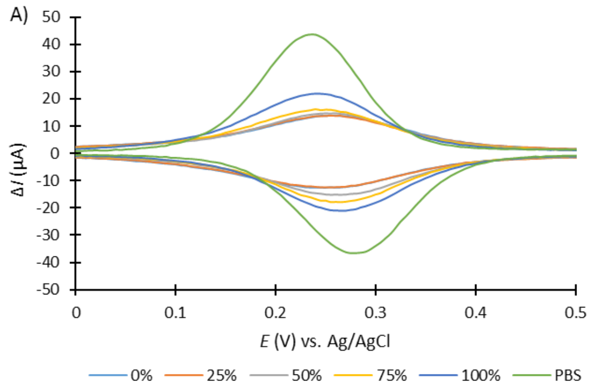

Figure S19 Effect of MCF-7/WGA percentage in test solution on DPV.
DPV signals obtained with a 2mm Au-RDE in 2.5mM ferrocyanide/2.5mM ferricyanide/1X PBS after rotation assisted adsorption at 2000rpm for 5 minutes in varying % methylation solutions. Methylated and unmethylated DNA derived from amplicons of the EN1 gene promoter from MCF-7 and WGA DNA respectively. Secondary PCR product of amplicons total 1/18 in solution. Mean ±SD (n=3).

Table S18 Effect of MCF-7/WGA percentage in test solution on peak current.
i_{pa} (µA) obtained with a 2mm Au-RDE in 2.5mM ferrocyanide/2.5mM ferricyanide/1X PBS after rotation assisted adsorption at 2000rpm for 5 minutes in varying % methylation solutions. Methylated and unmethylated DNA derived from amplicons of the EN1 gene promoter from MCF-7 and WGA DNA respectively. Secondary PCR product of amplicons total 1/18 in solution. Mean ±SD (n=3).

% Methylation	i_{pa} (µA)		
	Mean	SD	RSD (%)
PBS	43.61	0.25	0.58
0	13.90	0.18	1.32
25	13.87	0.15	1.10
50	14.74	0.57	3.85
75	16.21	0.45	2.75
100	21.94	0.53	2.42
